# Impact of concurrency on the performance of a whole exome sequencing pipeline

**DOI:** 10.1186/s12859-020-03780-3

**Published:** 2021-02-09

**Authors:** Daniele Dall’Olio, Nico Curti, Eugenio Fonzi, Claudia Sala, Daniel Remondini, Gastone Castellani, Enrico Giampieri

**Affiliations:** 1 Department of Physics and Astronomy, University of Bologna, 40127 Bologna, BO Italy; 2 Department of Experimental, Diagnostic and Specialty Medicine, University of Bologna, 40138 Bologna, BO Italy; 3 Istituto Scientifico Romagnolo per lo Studio e la Cura dei Tumori (IRST) IRCCS, 47014 Meldola, Italy

**Keywords:** Concurrency, Parallel computing, Bioinformatics, Analysis pipeline, Scalability, Efficiency, Workflow management system, Snakemake

## Abstract

**Background:**

Current high-throughput technologies—i.e. whole genome sequencing, RNA-Seq, ChIP-Seq, etc.—generate huge amounts of data and their usage gets more widespread with each passing year. Complex analysis pipelines involving several computationally-intensive steps have to be applied on an increasing number of samples. Workflow management systems allow parallelization and a more efficient usage of computational power. Nevertheless, this mostly happens by assigning the available cores to a single or few samples’ pipeline at a time. We refer to this approach as *naive parallel* strategy (NPS). Here, we discuss an alternative approach, which we refer to as *concurrent* execution strategy (CES), which equally distributes the available processors across every sample’s pipeline.

**Results:**

Theoretically, we show that the CES results, under loose conditions, in a substantial speedup, with an ideal gain range spanning from 1 to the number of samples. Also, we observe that the CES yields even faster executions since parallelly computable tasks scale sub-linearly. Practically, we tested both strategies on a whole exome sequencing pipeline applied to three publicly available matched tumour-normal sample pairs of gastrointestinal stromal tumour. The CES achieved speedups in latency up to 2–2.4 compared to the NPS.

**Conclusions:**

Our results hint that if resources distribution is further tailored to fit specific situations, an even greater gain in performance of multiple samples pipelines execution could be achieved. For this to be feasible, a benchmarking of the tools included in the pipeline would be necessary. It is our opinion these benchmarks should be consistently performed by the tools’ developers. Finally, these results suggest that concurrent strategies might also lead to energy and cost savings by making feasible the usage of low power machine clusters.

## Background

In this paper we examine multiple samples execution strategies which a standard bioinformatics pipeline can be run with, in order to determine a time-effective strategy that can be generally suggested to the bioinformatics community. We ideally aim at a processors usage setting that achieves the best speedup of the total execution time when multiple samples should be examined simultaneously. Computational strategies to obtain effective speedup for individual bioinformatics tools, such as BWA [1] and SAMtools [2], have been proposed [3, 4] and it is well-known that different strategies impact differently on the execution time of a single tool. Here, we are interested in studying how different strategies impact on the execution of whole bioinformatics pipelines. Our main focus is then optimal computational power (number of processors) allocation among the steps of the samples’ pipelines, whereas we consider all others machine resources, such as memory usage, cache and disk I/O operations, as boundaries that limit the highest possible performance that could be obtained.

A next-generation sequencing (NGS) data analysis pipeline involves several computationally-intensive tasks (like read trimming, alignment, BAM post-processing, etc...). Some of these tasks are non-scalable (NS), i.e. take a fixed amount of time when given multiple resources, and others are parallelly computable (PaCo), i.e. improve when given multiple resources. Each task is usually handled by a single specific tool, which might be optimized for parallel computing. Typically the tools demanding more computational time are implemented as PaCo in order to try to reduce the execution time. Therefore, most of the execution time in a typical pipeline is spent performing PaCo tasks. In addition, these pipelines are usually applied to several samples at once, and some steps need to be repeated for each sample while some are shared among them.

At first, pipelines were developed for a command line interpreter execution, i.e. UNIX shell. They were typically built as a sequential series of commands that needed to be reproduced for every sample. Nowadays many bioinformaticians [5–7] rely on workflow management systems (WMSs) [8–11] to improve execution efficiency and scalability. WMSs usually accept different programming languages within the same pipeline and can be run by a single command. Further, WMSs are designed to independently organize all pipeline tasks before the actual execution. They trace a graph of tasks by combining several factors, including expected results, existing data and dependencies. This feature enhances scalability and it is especially useful for mutually independent tasks, which can be run simultaneously without any explicit competence of parallel computing coding. In addition, many of these systems have options to indirectly manage machine resources.

Although WMSs allow to easily manage the execution of a pipeline for multiple samples, usually each sample’s pipeline is processed one at a time sequentially, even if in a highly optimized way. Indeed, the most basic way to run a pipeline for multiple samples is to implement it with one of these systems and launch it on a high-performing machine by setting the number of exploitable processors and letting the WMS assign the resources to each sample’s task. Often the default setting of WMSs is to allocate all the available processors for each step of any sample’s pipeline, unless otherwise specified. We define this parallel single sample strategy as naive parallel strategy (NPS). It is well-recognized that all PaCo tasks have a sub-linear increase of speedup (where linear is the most optimistic case, called “embarrassingly parallel”), which means they can not achieve unlimited boosting in their performances. This leads to a waste of resources since a PaCo task occupies a number of processors that it can not entirely exploit.

We observe that both the old sequential and the NPS follow a single-sample focus, while there is a compelling need to efficiently analyse large datasets, where the single-sample focus might lead to inefficiencies due to the PaCo sub-optimal parallelization. NPS, thanks to WMSs, can boost analyses in terms of automatic replicability and overall management simplification, but they have several limitations: they are not capable of predicting the scalability of PaCo tasks and they do not estimate either time or resources needed by any tasks.

With this paper, we question the usage of NPS and suggest a new execution strategy that we refer to as concurrent execution strategy (CES). To improve the usage of all processors, CES divides them equally among all samples’ pipelines. In doing so, samples’ pipelines can concur simultaneously and, though individually running slower, they can be completed at the same time all together, resulting in a lower total execution time. Moreover, CES implicitly tries to minimize the impact of sub-linearity of PaCo tasks on the overall total execution performance, which makes it even more suitable for pipelines that are heavily built around PaCo tasks. CES is then based on the idea of equally splitting the number of processors over samples’ pipelines. This strategy is not guaranteed to be the most efficient one in all occasions, but it is the simplest representation of a concurrent organization.

To empirically assess the efficacy of the CES compared with NPS we tested these two strategies on a WES pipeline that we have developed in our lab (detailed in “Methods” section).

**Fig. 1:**
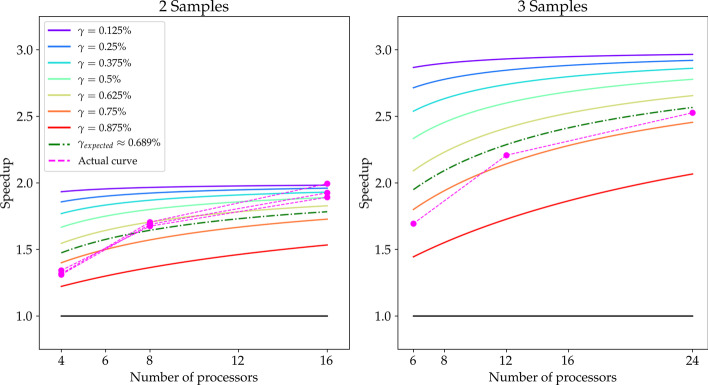
Speedup comparison. Expected speedup for CES compared to the NPS for 2 and 3 samples as a function of the available number of processors and PaCo proportion $\gamma$. The magenta dashed line represents the measured speedup of the real pipeline tested. The green dashed-dotted line represents the expected performance gain based on the estimated proportion of sequential execution time of our pipeline. In the 2-samples figure there are three observed speedups associated to all the combinations of two samples out of the three available. In the 3-samples figure there is a single observed speedup associated as there is only one possibile triplet out of the three available samples

**Fig. 2:**
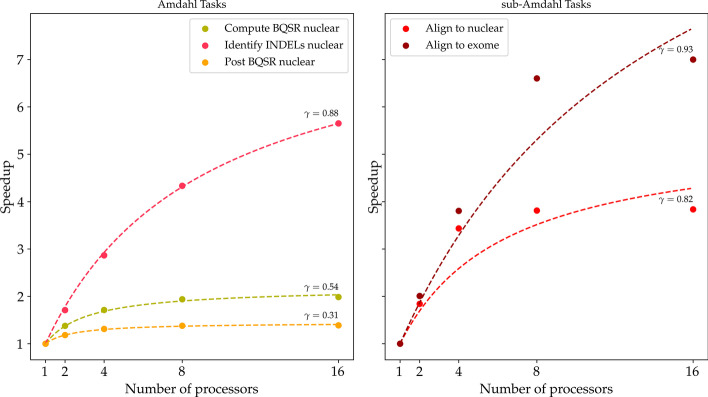
Single tasks’ speedup comparison. Performance gain of several PaCo tasks with respect to the number of processors exploited and the comparison with the Amdahl’s law predictions. The points represent the actual measured speedup. The dashed-lines shows the best Amdahl’s fit with the associated $\gamma$. On the left plot we report tasks behaving according to Amdahl’s law. On the right plot we report tasks with significant departure from Amdahl’s law predictions, with a speedup for high number of processors lower than expected. Only PaCo tasks with a significant speedup behaviour are reported

## Results

### Application of Amdahl’s law to identical processes

To study the performance of the CES we represent the execution of a pipeline for multiple samples in a mathematical framework. There are two levels that are worth to examine: the single-task level and the pipeline level. These levels are similar and closely related, since the latter is the combination of all tasks. For the sake of simplicity, we describe the mathematical framework of the former level in order to generalize it to the latter, which our work is focused on.

The single-task level describes the behaviour of those pipeline’s tasks that are PaCo. We assume these kind of tasks to be always composed by a NS component and an embarrassingly parallel (EP) component (Amdahl’s law hypothesis). The former does not change its execution time regardless of the number of processors allocated to the task. On the contrary, the latter will have a speedup in the execution time proportional to the number of allocated processors. Then, we define the total time *T* needed by a single-processor to process a single sample as:

1 $$\begin{aligned} T = F + P, \end{aligned}$$

where *F* and *P* represent execution times respectively for the NS component and EP component. We can also write the former equation, defining $\gamma = \frac{P}{T}$, as:

2 $$\begin{aligned} T = (1 - \gamma ) T + \gamma T , \end{aligned}$$

where $F=(1-\gamma )\,T$ and $P=\gamma \,T$. The value of $\gamma$ ranges from 0 to 1 and it stands for proportion of time of the EP task.

Now, Amdahl’s law [12] states that potential speedup in latency using *K* processors at fixed workload can be formulated as:

3 $$\begin{aligned} s(K, \gamma ) = \frac{1}{1 - \gamma + \frac{\gamma }{K}} . \end{aligned}$$

That is, a single-sample execution can be sped up with a factor equals to $s(K; \gamma )$ by supplying the process with *K* processors.

Next, we analyse the performances of both the NPS and CES when we need to run a PaCo task for *N* samples with *K* available processors. We assume for simplicity *K* is a *N* multiple. It is obvious to conclude that both strategies are better than a sequential execution.

With simple considerations we can describe execution time for both strategies respectively with

4 $$\begin{aligned} T_{NPS} \approx N \, \left( F + \frac{P}{K} \right) \end{aligned}$$

and

5 $$\begin{aligned} T_{CES} \approx \left( F + \frac{P}{ \left( \frac{K}{N} \right) } \right) . \end{aligned}$$

Using NPS the total time $T_{NPS}$ is composed of the sequential execution of the *N* samples each one at their best possible speed. Using CES all the processes of each sample are executed at the same time (but with less available processors) and thus the total time $T_{CES}$ would be the maximum execution time of each individual sample. All samples assumed equal, this becomes the execution time of a single sample using the allocated number $\frac{K}{N}$ of processors.

The same equations can be expressed in terms of $\gamma$ as:

6 $$\begin{aligned} \begin{aligned} T_{NPS} \approx \frac{N \, T}{K} \, [ \gamma \, (1-K) + K ] \\ T_{CES} \approx \frac{T}{K} \, [ \gamma \, (N-K) + K ] . \end{aligned} \end{aligned}$$

In analogy with Amdahl’s law, we can derive the speedup between the two strategies:

7 $$\begin{aligned} s(K, \gamma ) = \frac{ N \, [ \gamma \, (1-K) + K ] }{ \gamma \, (N-K) + K }. \end{aligned}$$

With $N>1$, speedup ranges from 1 ($\gamma = 1$) to *N* ($\gamma = 0$) with any *K* processors. Consequently, this result suggests that at the single-task level the CES is always more efficient than the NPS. This result is based on the assumption that the EP component has a linear and unbounded potential speedup. This assumption is not always true and many bioinformatics tools reach a plateau after a linear-wise increase, before one would expect from Amdahl’s law. Therefore the effective speedup of CES over NPS can in general be even higher than the expected one.

To understand how the CES speedup actually results at the single-task scale we take the example of BWA MEM tool, which is a widely used aligner that leverages on a seed-and-extend approach to align a sample to a reference sequence. For each read BWA MEM detects the perfect matches between the read subsets and the reference. Next, these matching subsets (seeds) are extended to the whole read, in order to assess the accuracy of each alignment. Eventually, the most accurate alignment for every read is kept. Seed detection and extension are the main PaCo tasks of the tool, whereas reading and writing files to the disk are the NS tasks. If we neglect the I/O bottleneck of disk operations, these NS tasks always take a fixed amount of time *F* for every sample in order to read the input reads and to write the output aligned reads. Therefore, the NPS takes $F \times N$ samples to read all input and write all output files, whereas the CES only takes *F* by performing all input and output operations altogether. In contrast, seed detection and extension are PaCo and, while the NPS runs these two tasks individually for each sample at their best speed, the CES runs them at lower speed but it processes all samples simultaneously. The execution time taken by PaCo tasks is equal for both strategies if they are considered EP but, since this is never the case in real executions, the NPS is overall slower than the CES for the execution of BWA MEM.

### CES speedup at single-task level on real data

We ran our pipeline on WES data of three public human paired-matched samples, one blood sample and one matching tumour. In Table 1 we report the average speedup of CES over NPS within pipeline executions for all PaCo tasks taking more than 15 min. We see the speedup for BWA MEM, underlying *Align to nuclear* and *Align to exome*, to be always greater than 1 as well as for all the other PaCo tasks.

**Table 1:** Average speedup of CES over NPS for all PaCo tasks taking on average at least 15 min

Task	4 processors	8 processors	16 processors
	Mean ± Std (s)	Mean ± Std (s)	Mean ± Std (s)
(a) 2-samples execution			
Align to nuclear	1.75 ± 0.01	2.84 ± 0.02	3.19 ± 0.04
Align to exome	1.76 ± 0.01	1.79 ± 0.01	2.87 ± 0.07
Identify INDELs nuclear	1.95 ± 0.01	1.83 ± 0.01	2.15 ± 0.09
Compute BQSR nuclear	2.38 ± 0.03	2.69 ± 0.02	2.91 ± 0.15
Post BQSR nuclear	3.09 ± 0.01	3.17 ± 0.03	3.1 ± 0.04

Task	6 processors	12 processors	24 processors
	Mean ± Std (s)	Mean ± Std (s)	Mean ± Std (s)
(b) 3-samples execution.			
Align to nuclear	2.05 ± 0.01	3.72 ± 0.01	3.91 ± 0.05
Align to exome	1.61 ± 0.01	2.06 ± 0.01	3.62 ± 0.05
Identify INDELs nuclear	1.88 ± 0.03	2.2 ± 0.06	2.75 ± 0.16
Compute BQSR nuclear	2.96 ± 0.02	3.26 ± 0.03	3.96 ± 0.14
Post BQSR nuclear	4.32 ± 0.03	4.37 ± 0.12	4.43 ± 0.04

Number of processors stands for the number of available processors managed by both strategies

Since we are not focusing on the single-task level but on the pipeline level, we extend the previous analytic procedure to look at NPS and CES when we need to run a whole bioinformatics pipeline for *N* samples simultaneously with *K* available processors. We can assume that a bioinformatics pipeline can be represented by Eq. 1 as well, since it is a combination of several NS and PaCo tasks. Therefore, we can generalize the previous result and claim that the CES does perform faster than the NPS at the pipeline-level as well.

### CES speedup at pipeline level on real data

We compared the execution time and memory occupation running a variants calling pipeline on the subjects either with the NPS and with the CES. The average execution time for pipeline’s tasks, for all processors configuration and for each strategy are reported in as Tables in Additional file 1.

We relate our results in terms of speedup to those expected by Eq. 7. In Fig. 1 we represent several theoretical speedup behaviours given different $\gamma$ values as continuous lines. Since we can estimate the improvable proportion of sequential execution time of our pipeline ($\gamma _{\text {expected}}$), Fig. 1 also shows the expected speedup for this value (as green dashdotted lines), which is about 0.689. To perform this estimate we approximated the pipeline’s PaCo tasks as EP.

Figure 1 clearly shows the CES to be faster than the NPS. Indeed, our actual speedups have values greater than 1 regardless of the number of processors and the number of samples. Besides, the CES completes hourly more tasks than the NPS, as shown in Table 2.

**Table 2:** Summary statistics of completed tasks per hour (median, lower and upper quartiles are reported)

Processors	NPS	CES
	Median (Q1–Q3)	Median (Q1–Q3)
(a) 2-samples execution		
4	3 (1–5)	4.5 (2–10)
8	4 (2–6)	9 (4–12)
16	3 (2–8)	7.5 (4–16.5)

Processors	NPS	CES
	Median (Q1–Q3)	Median (Q1–Q3)
(b) 3-samples execution		
6	3 (2–6)	6 (2–15)
12	4 (2–5)	16.5 (4–21.75)
24	3 (2–4)	13.5 (8.25–18)

Only tasks taking at least 1 min are considered. Number of processors stands for the number of available processors managed by both strategies

Nonetheless, curves in Fig. 1 do not accurately fit the theoretical curves that are expected by $\gamma _{\text {expected}}=0.689$. It should be noted that the PaCo tasks are not EP as it was assumed during the estimate of $\gamma _{\text {expected}}$. Hence, the actual value of $\gamma _{\text {expected}}$ is probably lower.

In general, we notice that our observed speedup behaviours increase faster than expected by Eq. 7. This can be explained by the fact that in real executions the overall speedup produced by the pipeline’s PaCo tasks follows a sub-Amdahl increase, since PaCo tasks performances improve sub-linearly. The sub-Amdahl speedup increase, showed for some PaCo tasks in Fig. 2, expresses the loss of performance along the number of supplied processors.

Therefore, it is more efficient to run samples’ pipelines with processors evenly distributed, than to run them with all processors assigned to each sample’s pipeline one after the other. This aspect becomes progressively more evident when many processors are involved in the execution: we observe from all speedups shown in Fig. 2 that there are almost no differences in speedup by using 8 and 16 processors, which suggests a waste of resources.

**Fig. 3:**
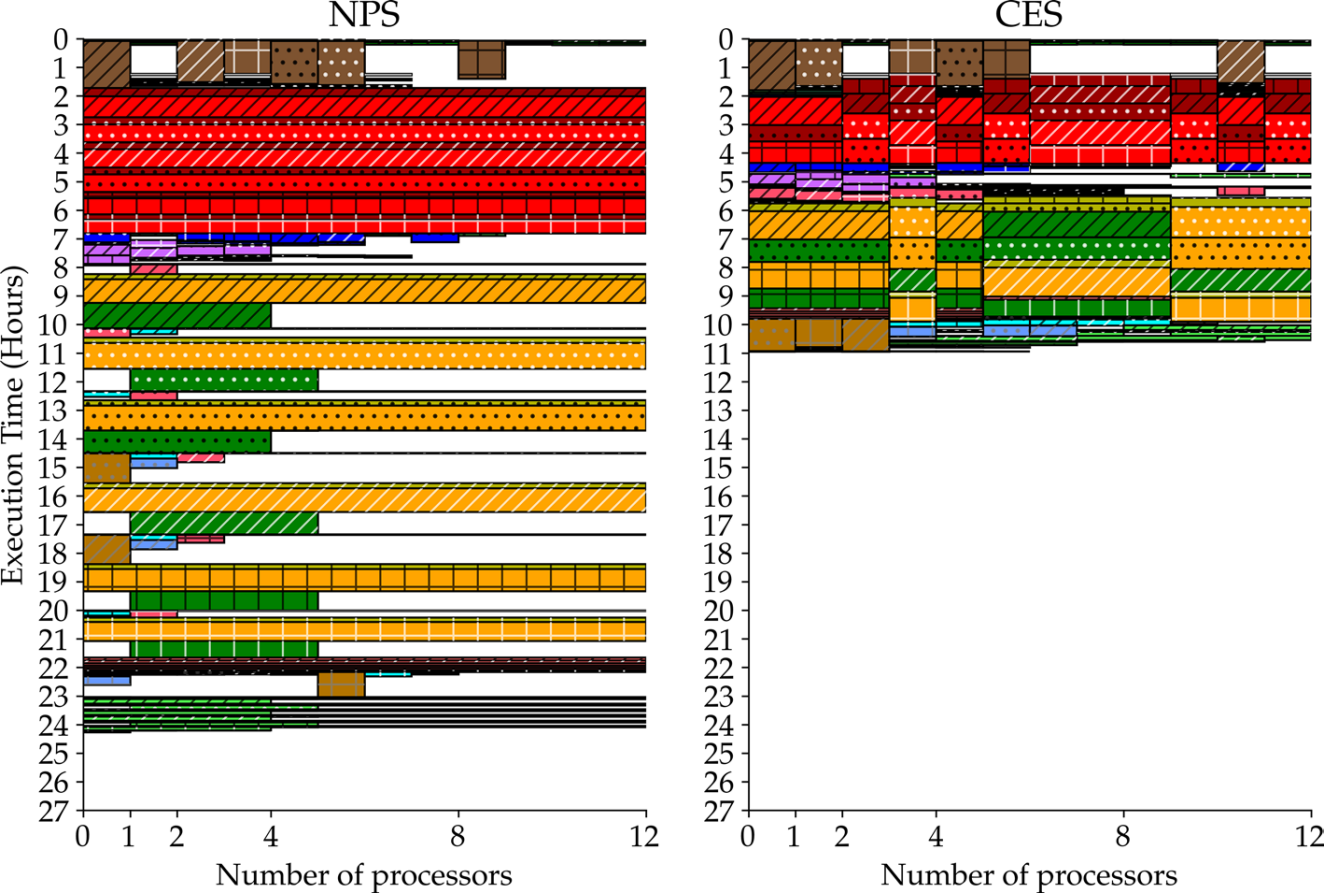
Pipelines execution comparison (processors allocation). Representation of a pipeline execution on all 3 samples based on processors usage along time for both NPS and CES. Each rectangle represents a task, its base and height are respectively equal to the allocated processors and to the time elapsed. The hatch type indicates the paired-matched sample that a task works on. The hatch color specifies the data type taken as input by a task. The color identifies the task type. See Fig. 5 for comprehensive legend. See Fig. 4 for its corresponding memory usage

The lack of agreement between actual and theoretical increase in speedup can be also explained by the CES and NPS tasks and processors subdivision. Figure 3 shows both subdivisions along time for a 3-samples case that has been run with 12 processors.

We notice from Fig. 3 that NS components are actually run as soon as the necessary resources are available. The NPS is then penalized by Eq. 4 because it actually does not need to repeat all NS tasks *N* times and the fixed time component (*F*) is less than what assumed. That is, our actual speedup can be lower than expected. Nonetheless this reduction is small because the NPS is still affected by a slowdown compared to the CES, since the PaCo tasks allocate all processors ending up being executed sequentially (see NPS panel in Fig. 3). In contrast, the CES is slightly penalized by Eq. 5, given that the NS component is actually less than what assumed.

**Fig. 4:**
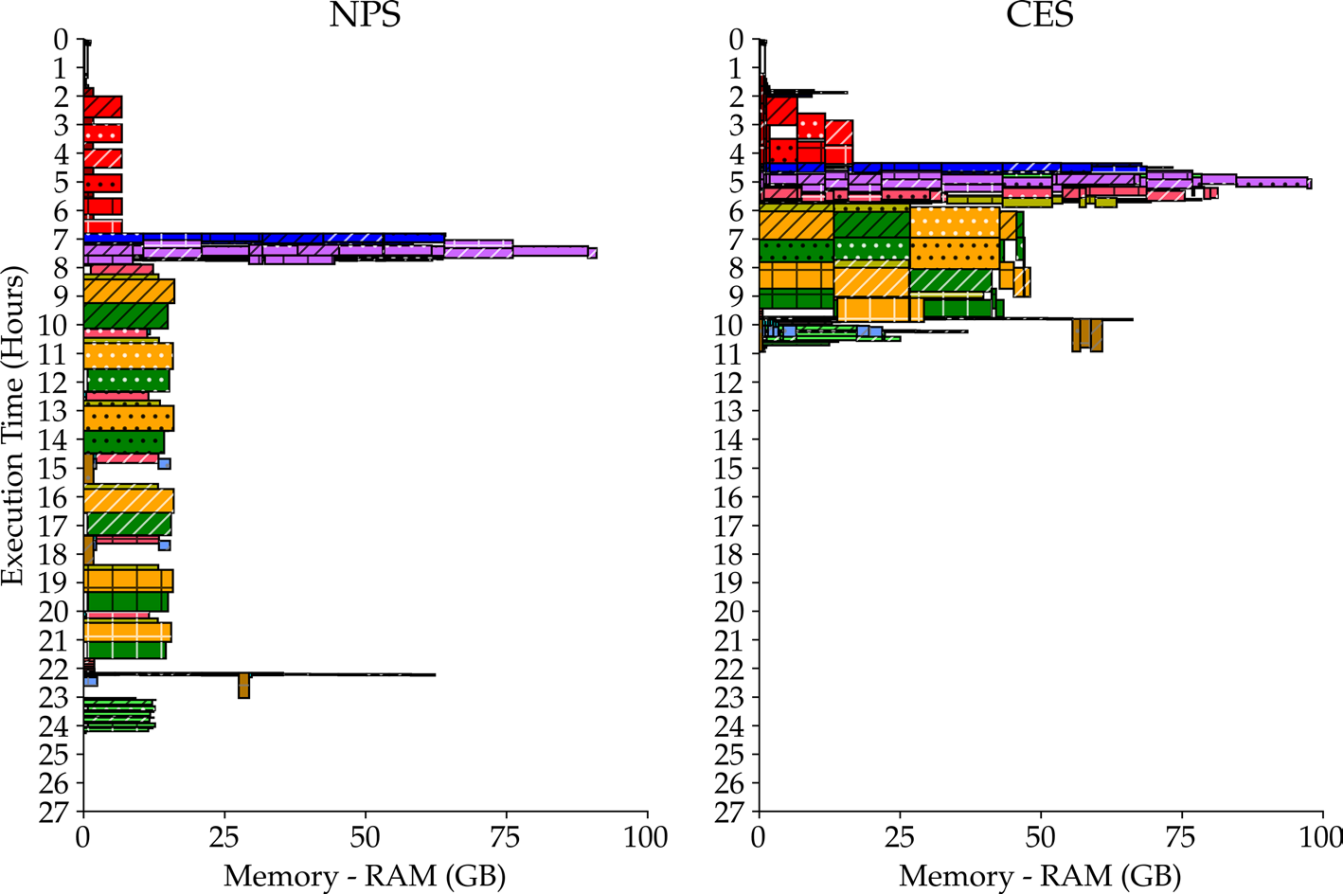
Pipelines execution comparison (memory usage). Representation of a pipeline execution on all 3 samples based on memory usage along time for both NPS and CES. Each rectangle represents a task, its base and height are respectively equal to the allocated amount of memory and to the time elapsed. The hatch type indicates the paired-matched sample that a task works on. The hatch color specifies the data type taken as input by a task. The color identifies the task type. See Fig. 5 for comprehensive legend. See Fig. 3 for its corresponding processors usage

Furthermore, we observe from Fig. 1 two different behaviours respectively for the 2-samples case and the 3-samples one. In the former, the observed speedups are lower than expected but they increase until exceeding it, whereas in the latter the observed speedup approaches the expected one but it is always below it. This difference can be explained by looking at the memory usage of both cases, reported in Fig. 4 analogously to Fig. 3. In the 3-samples case we notice a memory overloading problem in a middle step of the pipeline between 4th and 6th hours of execution. We do not have the same limit with the 2-samples case, where we run our pipeline as if the available memory is unbounded (see Additional file 5). We can then assume that the actual speedup in the 3-samples case would exceed the theoretical one as the 2-samples case speedups do, but this does not happen due to technical limitations.

All Figures for other processors and memory usage cases (2-samples and different number of processors) are reported in Additional files 2–5.

## Discussion

Nowadays, many researchers working in the bioinformatics field design and implement pipelines for the processing and analysis of biological data. Different execution strategies of these pipelines can lead to considerable differences in execution times. In this paper we discuss two strategies which a bioinformatics pipeline can be run with when a multiple samples execution is required: NPS and CES. Our objective was to determine the best strategy among these two approaches in a general case of running a whole bioinformatics pipeline for *N* samples simultaneously and with at most *K* available processors. As explained above, each strategy consists respectively in:

- running the pipeline for all samples simultaneously by allowing each sample’s pipeline to potentially allocate all *K* processors (NPS), this is the standard approach for WMSs;
- running the pipeline for all samples simultaneously by equally splitting *K* processors across samples’ pipelines (CES).

We expressed mathematically that the CES is always the most efficient strategy, when there are no other technical limitations, and we did measure it with actual executions on a WES pipeline developed in our lab (detailed in “Methods” section).

Although we examined a simple case of CES, we obtained performance gains in terms of speedup up to 2–2.4 with respect to the NPS. These gains are fairly consistent with the single-task level expectations given by Eq. 7 that we projected onto the pipeline-level. Nevertheless, several aspects need always to be addressed, as suggested by our results, since stepping from a single-task level to the pipeline level is not completely straightforward.

First, Eqs. 4 and 5 still consider an optimistic linear speedup for all PaCo steps. As shown in Fig. 2, PaCo tasks are known to follow a speedup lower than the one expected from Amdahl’s law with respect to supplied processors. Then the CES over NPS speedup could be expected to reach greater values. Indeed, by limiting the processors of the PaCo tasks, the CES reduces the overall waste of resources given by the sub-linear scaling of such tasks. We expect this advantage to play an instrumental role in the near future since bioinformatics pipelines are progressively being developed around PaCo tasks.

Secondly, both equations are obtained assuming the NS component to be either a single step or a series of sequential steps. As we saw in Fig. 3, none of these assumptions is true at pipeline level because all samples’ NS components are mutually independent. This fact implies that samples’ NS components can generally be run as soon as necessary resources are available, resulting in a machine usage optimization and eventually in an execution time reduction for both strategies. Therefore, we generally can expect a speedup decrease when the organization of the NS components is similar between the NPS and the CES (to see an example of this effect observe the first 2 hours of execution with both strategies in Fig. 3). This happens when the NPS slowdown compared with CES does not hinder significantly the whole execution. Otherwise, we expect an increase in speedup, since the CES always manages to optimize available resources, whereas the NPS tends to focus them only on few tasks. These expectations are confirmed by the results shown in Fig. 1, where the speedup is lower than expected when using few processors (4 and 6 respectively with 2 and 3 samples) and it increases when the number of processors is increased, approaching the expected values.

Third, the use of the other machines resources at the pipeline level, such as memory usage, cache and disk I/O operations, can be much heterogeneous across tasks, which means it is likely that above a certain number of samples one of them will cause a bottleneck at some point of the execution. In our experimentation we discovered a memory bottleneck for the 3-samples case which interfered with the performance of CES. Thus, a general *N* samples execution on a *K* cores machine is actually affected by shared and limited amount of memory, cache and disk I/O speed. Bottlenecks of such kind, which are pipeline and machine dependent, are expected to bound the number of samples that can be executed concurrently limiting the theoretical increase of CES speedup. It is noteworthy, though, that alongside a reduction in latency and a smart managing of processors, the CES also accidentally improves memory usage. In fact, as can be seen in Fig. 4 the NPS underutilizes the available memory, while the CES has less unused resources throughout its execution.

Fourth, Eqs. 4 and 5 are true when all *N* samples share the same execution time *T*, which is a very rough approximation since this quantity depends necessarily on the input data size. If *T* across samples is roughly uniform, i.e. same order of magnitude, then the previous approximation is reliable. When this condition is not met, the CES does not always guarantee to be faster than the NPS because the execution time becomes the time needed by the largest sample. Besides, in such cases, the CES risks to waste a lot of machine resources when all small samples end their run and only the largest sample still has to complete. Thus, properly tailored concurrency strategies need to be designed when samples input size variance is large. To this end, instead of evenly distributing processors, an effective improvement could be achieved by a concurrent strategy that allocate processors based on input size relative to the other inputs, i.e. largest samples get more processors. Usually samples are acquired by the same experimental design and therefore they are roughly of the same size. This was true for our 3 samples, so that we could test the CES without the need of more advanced strategies.

**Fig. 5:**
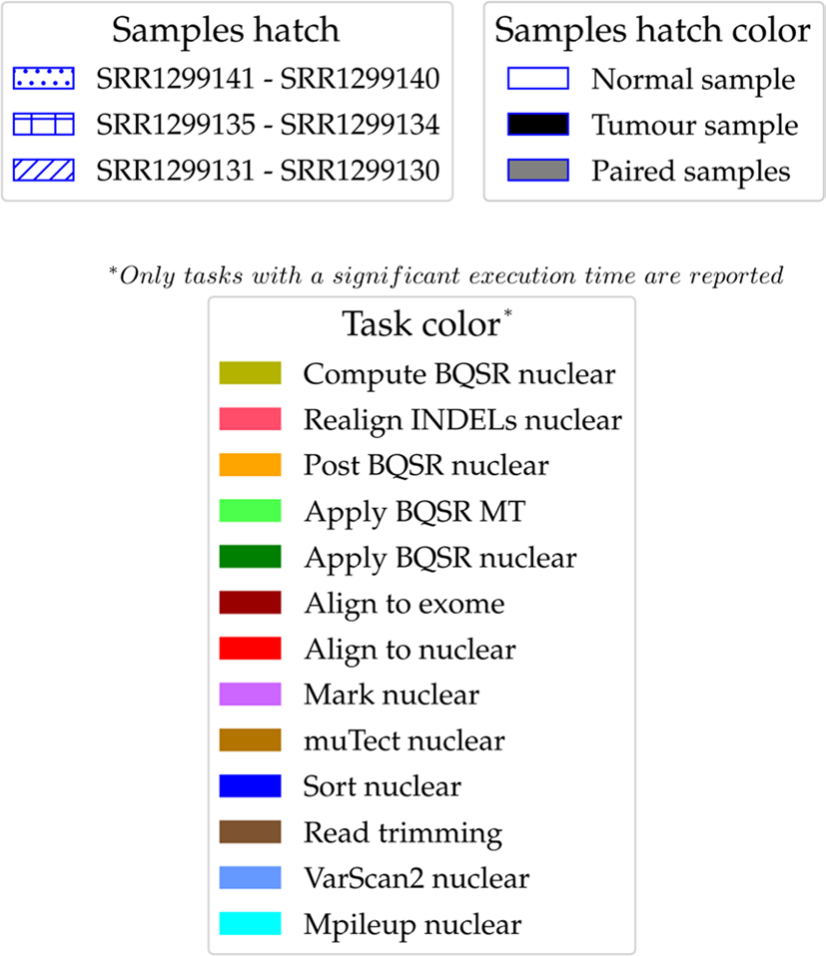
Legend for Figs.3 and 4

## Conclusions

With this paper we described a general approach, the CES, to run bioinformatics pipelines for multiple samples that is alternative to the NPS and might be followed to boost performances. Performance gains are achieved by a strategy based on the idea of limiting the individual resources assigned to each sample’s pipeline and to let them concur. With a bioinformatics pipeline developed in our lab we found CES speedups over NPS up to 2–2.4. We noticed that more complex *concurrent* schemes may yield faster executions; though this would require to measure all pipeline’s tasks behaviours and to have a deep knowledge of machine resources (processors, memory, cache and disk I/O speed) in order to find an optimal distribution. Since most of such tasks use tools developed by specialized companies, these may aid bioinformaticians by providing documentation about speedups as a function of number of processors and input data size, alongside memory usage and disk access.

These findings have implications for the feasibility of low-power clusters to be used in bioinformatics analyses, as the division of resources has not to necessarily occur on the same machine but on multiple ones. Since CES limits each task to relatively few resources, one can potentially distribute all tasks across multiple machines with lower requirements in terms of resources, such as low-power machines. We did not thoroughly analyse this possibility, but in some preliminary analysis we observed no significant difference to occur between using a cluster of low-power machines and a single high performing machine server [13]. The main benefits of this alternative are the noteworthy saving on electrical costs and the chance to acquire a greater amount of resources with multiple low power machines for the same cost of a single traditional server. The feasibility of running bioinformatics pipelines on low-power machine clusters with CES is yet to be fully explored, but might potentially offer the chance to time-effective executions alongside relevant savings.

## Methods

### WES pipeline

We decided to exploit a WMS called Snakemake [8], which adapts the GNU Make concept reimplemented leveraging Python language. We chose this system due to its ability of handling intermediate files, its rich options set and its user friendliness. Furthermore, Snakemake is able to use the Conda environment management system [14] both for creation and activation of several environments at runtime.

Our pipeline follows the typical steps for somatic variants calling from WES data and it has been validated with specific version of tools, most of which are provided automatically by Conda (see Additional file 6: Table 5). After preliminary trimming (Adapter-Removal) [15, 16], reads are mapped to the selected reference human genome with BWA MEM [1]; resulting BAM are post-processed with tools from SAMtools, Picard [2, 17] and GATK (sorting, indexing, marking of duplicates, indel realignment, base quality score recalibration) [18]. Indels and SNVs are then independently called with MuTect [19] and VarScan2 [20], annotated with ANNOVAR [21] and filtered with in-house scripts. Additionally, this pipeline is also able to call mitochondrial variants: trimmed reads are independently aligned to a reference exome and those that aligned off-target are collected (Picard) and re-aligned to the mitochondrial genome. From this point, BAM post-processing, variant calling, annotation and filtering are executed as for the nuclear genome variants. Further, our pipeline benchmarks everyone of its steps thanks to Snakemake, which relies on *psutil* to retrieve information about each task, such as execution time and memory usage.

### Input data

WES FASTQ files were downloaded from NCBI’s Sequence Read Archive (SRA) [22]. They derive from three pairs of matched tumour-normal GIST samples (SRR1299130–SRR1299131, SRR1299134–SRR1299135, SRR1299140–SRR1299141), whose genomic DNA was enriched by exome capture with SureSelect Human All Exon 50Mb kit (Agilent Technologies) and sequenced on the Illumina HiSeq 2000 platform in 100-bp paired-end reads. Digital size ranges from 4.1GB to 5.7GB.

### Tested executions

We tested the following processors configurations by using Snakemake’s *cores* option. At first we sequentially run our pipeline on all samples by first supplying 2, then 4 and 8 processors.

Afterwards we organized our three samples in the three possible pairs and we independently run each of them using both the NPS and the CES. We tested three processors settings: 4, 8 and 16 processors. Both strategies exploited the same number of available processors but, as previously explained, the latter strategy split this number equally over samples, whereas the former did not.

Lastly we compared the two strategies by running our pipeline on all three samples within the same execution. We still set up three processors configurations (6, 12, 24) and we independently used both strategies. We decided to complete the three pairs and triplet runs with several processors settings in order to accurately explore the mathematical domain of Eq. 7.

### Machine server technical specifications

We performed all our computations on a standard high-performance machine server equipped with two $Xeon\,\,E5-2620v4$ CPUs, $2\,TB$ of storage (HDD) and $128\,GB$ of memory (RAM). In detail, the mounted $Xeon\,\,E5-2620v4$ CPU consists of a Broadwell-EP microarchitecture with $2.10(3.00)\,GHz$ frequency, 8 cores and $20\,MB$ of cache.

## Supplementary information

**Additional file 1:** **Tables 1–4.** Average execution time for pipeline's tasks, for all processors configuration and for each strategy.

**Additional file 2:** **Figure 1.** Representation of all executions (6, 12 and 24 processors) on all 3 samples based on processors usage along time for both NPS and strategies (Fig. 3 as well).

**Additional file 3.** **Figure 2.** Representation of all executions (6, 12 and 24 processors) on all 3 samples based on memory usage along time for both NPS and strategies (Fig. 4 as well).

**Additional file 4.** **Figure 3.** Representation of all executions (4, 8 and 16 processors) on 2 samples based on processors usage along time for both NPS and strategies (all possible 2-samples combinations are reported).

**Additional file 5.** **Figure 4.** Representation of all executions (4, 8 and 16 processors) on 2 samples based on memory usage along time for both NPS and strategies (all possible 2-samples combinations are reported).

**Additional file 6.** **Table 5.** Description of the NGS pipeline used for this paper.

## Data Availability

The datasets supporting the conclusions of this article are available in the National Center for Biotechnology Information (NCBI) repository: SRR1299130 (https://www.ncbi.nlm.nih.gov/sra/?term=SRR1299130), SRR1299131 (https://www.ncbi.nlm.nih.gov/sra/?term=SRR1299131), SRR1299134 (https://www.ncbi.nlm.nih.gov/sra/?term=SRR1299134), SRR1299135 (https://www.ncbi.nlm.nih.gov/sra/?term=SRR1299135), SRR1299140 (https://www.ncbi.nlm.nih.gov/sra/?term=SRR1299140), SRR1299141 (https://www.ncbi.nlm.nih.gov/sra/?term=SRR1299141).

## Abbreviations

WGS: Whole genome sequencing
WMS: Workflow management system
NPS: Naive parallel strategy
CES: Concurrent execution strategy
PaCo: Parallelly computable
WES: Whole exome sequencing
GIST: GastroIntestinal stromal tumour
NGS: Next-generation sequencing
NS: Non-scalable
EP: Embarrassingly parallel
SRA: Sequence read archive
