# Correction to: Impact of concurrency on the performance of a whole exome sequencing pipeline

**DOI:** 10.1186/s12859-021-04205-5

**Published:** 2021-06-01

**Authors:** Daniele Dall’Olio, Nico Curti, Eugenio Fonzi, Claudia Sala, Daniel Remondini, Gastone Castellani, Enrico Giampieri

**Affiliations:** 1 Department of Physics and Astronomy, University of Bologna, 40127 Bologna, BO Italy; 2 Department of Experimental, Diagnostic and Specialty Medicine, University of Bologna, 40138 Bologna, BO Italy; 3 Istituto Scientifico Romagnolo per lo Studio e la Cura dei Tumori (IRST) IRCCS, 47014 Meldola, Italy

## Correction to: BMC Bioinformatics (2021) 22:60. 10.1186/s12859-020-03780-3

Following the publication of the original article [1], the authors identified that the funding note is incorrect.

The correct funding note is given below.

Funding note:

This project has received funding from the Innovative Medicines Initiative 2 Joint Undertaking under grant agreement No 116026 H2020 EU "HARMONY" project. This Joint Undertaking receives support from the European Union’s Horizon 2020 research and innovation programme and EFPIA. Moreover the research leading to these results has received funding from the European Union’s Horizon 2020 research and innovation programme “VEO” Grant n. 874735, and from ETN-ITN “ImforFuture” Grant n. 721815. The “HARMONY” and “VEO funding bodies were partially used to pay staff salaries, while the “ImforFuture” funding body partially supported the purchase of the high performing machine server used for the experiment.

The author group has been updated above and the original article [1] has been corrected.
