# Supplementary material for: Impact of concurrency on the performance of a whole exome sequencing pipeline
Source: BMC Bioinformatics. 2021 Feb 9;22:60. doi: 10.1186/s12859-020-03780-3 (PMC7874478; doi:10.1186/s12859-020-03780-3)
Supplement: Supplementary file 1 — Additional file 1: Tables 1–4. Average execution time for pipeline's tasks, for all processors configuration and for each strategy. [file 12859_2020_3780_MOESM1_ESM.pdf]

Average execution time for pipeline's tasks, for all processors configuration and for each strategy

Table 1: Summary of average execution times for each task for a 2-samples run with NPS. The reported amount of processors is the number of them supplied to the whole run and managed by the chosen strategy. Only tasks with at least 60 seconds of execution time have been reported. PaCo tasks are labelled by *.

Task	4 processors $Mean \pm Std\,(s)$	8 processors $Mean \pm Std\,(s)$	16 processors $Mean \pm Std\,(s)$
Read trimming	4739 ± 574	4740 ± 560	4737 ± 580
Align to nuclear*	2367 ± 270	2077 ± 207	2059 ± 208
Align to exome*	1589 ± 160	885 ± 90	804 ± 88
Align to MT*	275 ± 24	272 ± 23	274 ± 24
Sort nuclear	906 ± 112	1002 ± 122	1005 ± 120
Sort exome	278 ± 32	301 ± 34	301 ± 33
Sort MT	127 ± 13	135 ± 13	135 ± 13
Mark nuclear	1064 ± 101	1055 ± 94	1050 ± 89
Mark MT	125 ± 11	131 ± 12	131 ± 11
Index nuclear	134 ± 20	132 ± 15	125 ± 13
Realign INDELs nuclear*	989 ± 109	991 ± 104	985 ± 105
Realign INDELs MT*	175 ± 16	174 ± 16	175 ± 17
Compute BQSR nuclear*	861 ± 91	667 ± 62	602 ± 47
Apply BQSR nuclear	2636 ± 303	2676 ± 296	2678 ± 316
Apply BQSR MT	485 ± 56	482 ± 59	476 ± 56
Post BQSR nuclear*	3150 ± 350	2965 ± 345	2892 ± 309
MuTect nuclear	3367 ± 260	3392 ± 250	3384 ± 241
Mpileup nuclear	589 ± 56	595 ± 47	594 ± 48
VarScan2 nuclear	1122 ± 69	1124 ± 71	1119 ± 71

Table 2: Summary of average execution times for each task for a 2-samples run with CES. The reported amount of processors is the number of them supplied to the whole run and managed by the chosen strategy. Only tasks with at least 60 seconds of execution time have been reported. PaCo tasks are labelled by *.

Task	4 processors $Mean \pm Std\,(s)$	8 processors $Mean \pm Std\,(s)$	16 processors $Mean \pm Std\,(s)$
Read trimming	4712 ± 578	4855 ± 620	4907 ± 679
Align to nuclear*	4846 ± 526	2645 ± 281	2315 ± 245
Align to exome*	3263 ± 329	1770 ± 220	981 ± 134
Align to MT*	285 ± 24	300 ± 30	322 ± 34
Sort nuclear	907 ± 106	1019 ± 145	1041 ± 135
Sort exome	286 ± 34	317 ± 46	318 ± 37
Sort MT	128 ± 13	135 ± 13	148 ± 20
Mark nuclear	1028 ± 123	1135 ± 151	1122 ± 122
Mark MT	124 ± 12	136 ± 18	142 ± 19
Index nuclear	130 ± 18	145 ± 20	156 ± 25
Realign INDELs nuclear*	1035 ± 118	1102 ± 144	1167 ± 149
Realign INDELs MT*	182 ± 16	196 ± 20	207 ± 15
Compute BQSR nuclear*	1293 ± 153	890 ± 101	739 ± 78
Apply BQSR nuclear	3299 ± 351	2587 ± 310	2604 ± 292
Apply BQSR MT	599 ± 63	456 ± 45	453 ± 45
Post BQSR nuclear*	3664 ± 434	3361 ± 386	3143 ± 474
MuTect nuclear	3451 ± 318	3609 ± 246	3544 ± 273
Mpileup nuclear	597 ± 57	651 ± 63	651 ± 71
VarScan2 nuclear	1145 ± 79	1262 ± 73	1193 ± 75

Table 3: Summary of average execution times for each task for a 3-samples run with NPS. The reported amount of processors is the number of them supplied to the whole run and managed by the chosen strategy. Only tasks with at least 60 seconds of execution time have been reported. PaCo tasks are labelled by *.

Task	6 processors $Mean \pm Std\,(s)$	12 processors $Mean \pm Std\,(s)$	24 processors $Mean \pm Std\,(s)$
Read trimming	5186 ± 574	5300 ± 596	5367 ± 593
Align to nuclear*	2221 ± 277	2198 ± 279	2244 ± 288
Align to exome*	1216 ± 160	853 ± 109	887 ± 113
Align to MT*	295 ± 38	298 ± 40	303 ± 41
Sort nuclear	1003 ± 121	1040 ± 124	1047 ± 124
Sort exome	299 ± 35	306 ± 35	307 ± 35
Sort MT	144 ± 17	154 ± 18	153 ± 18
Mark nuclear	1127 ± 124	1162 ± 116	1159 ± 124
Mark MT	141 ± 14	147 ± 13	148 ± 13
Index nuclear	170 ± 34	173 ± 31	171 ± 32
Realign INDELs nuclear*	1041 ± 119	1044 ± 118	1050 ± 120
Realign INDELs MT*	186 ± 22	187 ± 22	187 ± 22
Compute BQSR nuclear*	741 ± 59	640 ± 45	595 ± 34
Apply BQSR nuclear	2807 ± 350	2723 ± 353	2703 ± 335
Apply BQSR MT	524 ± 75	485 ± 62	492 ± 66
Post BQSR nuclear*	3047 ± 283	2933 ± 274	2891 ± 243
MuTect nuclear	3465 ± 240	3541 ± 240	3538 ± 222
Mpileup nuclear	609 ± 54	623 ± 39	623 ± 41
VarScan2 nuclear	1120 ± 55	1138 ± 51	1140 ± 51

Table 4: Summary of average execution times for each task for a 3-samples run with CES. The reported amount of processors is the number of them supplied to the whole run and managed by the chosen strategy. Only tasks with at least 60 seconds of execution time have been reported. PaCo tasks are labelled by *.

Task	6 processors $Mean \pm Std\,(s)$	12 processors $Mean \pm Std\,(s)$	24 processors $Mean \pm Std\,(s)$
Read trimming	5176 ± 576	5350 ± 663	5749 ± 894
Align to nuclear*	5476 ± 689	3007 ± 373	2859 ± 348
Align to exome*	3757 ± 498	2045 ± 299	1229 ± 166
Align to MT*	319 ± 42	332 ± 41	359 ± 40
Sort nuclear	1019 ± 113	1038 ± 126	1213 ± 146
Sort exome	312 ± 49	321 ± 38	395 ± 44
Sort MT	138 ± 15	151 ± 17	163 ± 20
Mark nuclear	1136 ± 152	1154 ± 127	1253 ± 146
Mark MT	135 ± 15	149 ± 18	159 ± 24
Index nuclear	150 ± 24	161 ± 25	178 ± 21
Realign INDELs nuclear*	1182 ± 158	1221 ± 133	1274 ± 159
Realign INDELs MT*	203 ± 23	219 ± 31	236 ± 25
Compute BQSR nuclear*	1371 ± 129	1008 ± 102	811 ± 72
Apply BQSR nuclear	3558 ± 451	2740 ± 357	2811 ± 349
Apply BQSR MT	623 ± 80	503 ± 55	507 ± 70
Post BQSR nuclear*	3864 ± 359	3603 ± 346	3552 ± 330
MuTect nuclear	3686 ± 294	3936 ± 245	3863 ± 495
Mpileup nuclear	649 ± 59	722 ± 65	713 ± 67
VarScan2 nuclear	1206 ± 46	1328 ± 68	1307 ± 176
