# Supplementary figures and images for: Impact of concurrency on the performance of a whole exome sequencing pipeline
Source: BMC Bioinformatics. 2021 Feb 9;22:60. doi: 10.1186/s12859-020-03780-3 (PMC7874478; doi:10.1186/s12859-020-03780-3)

### 3 Samples with 6 processors

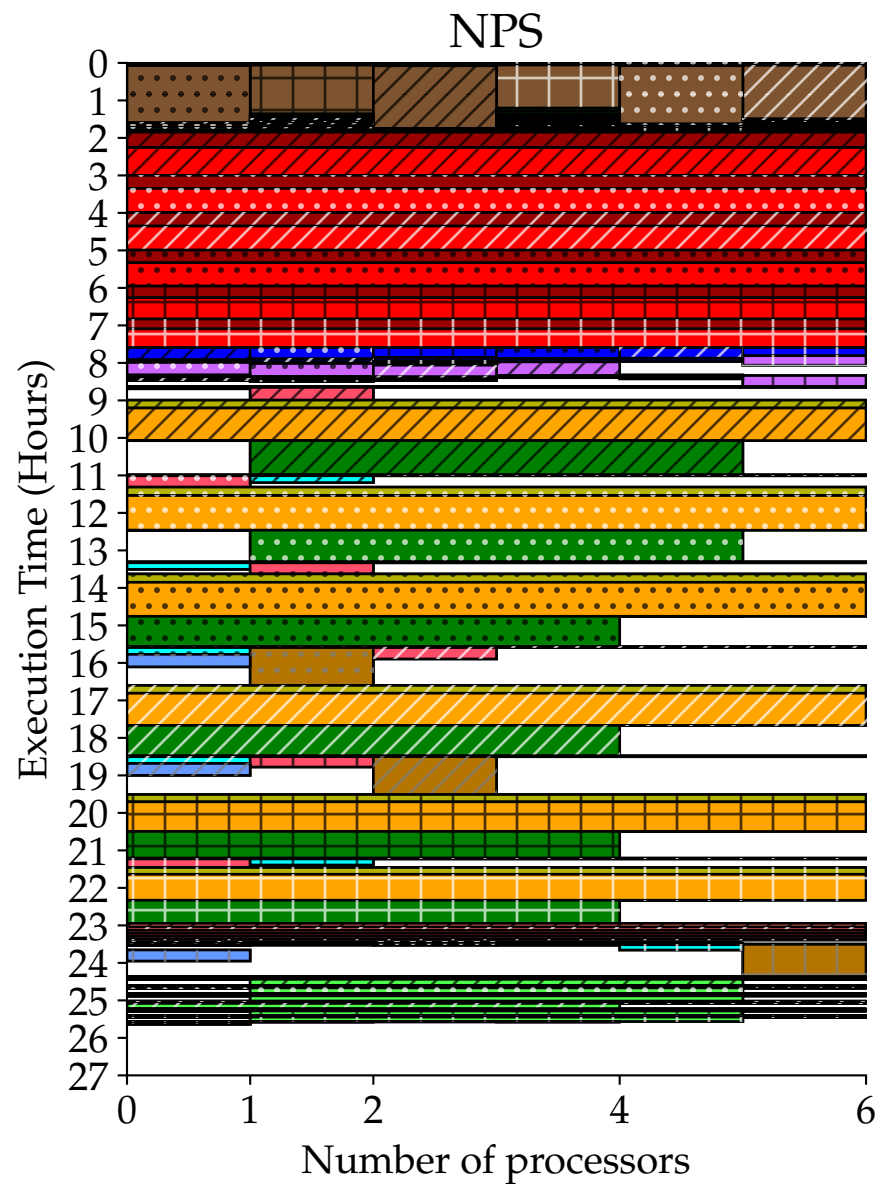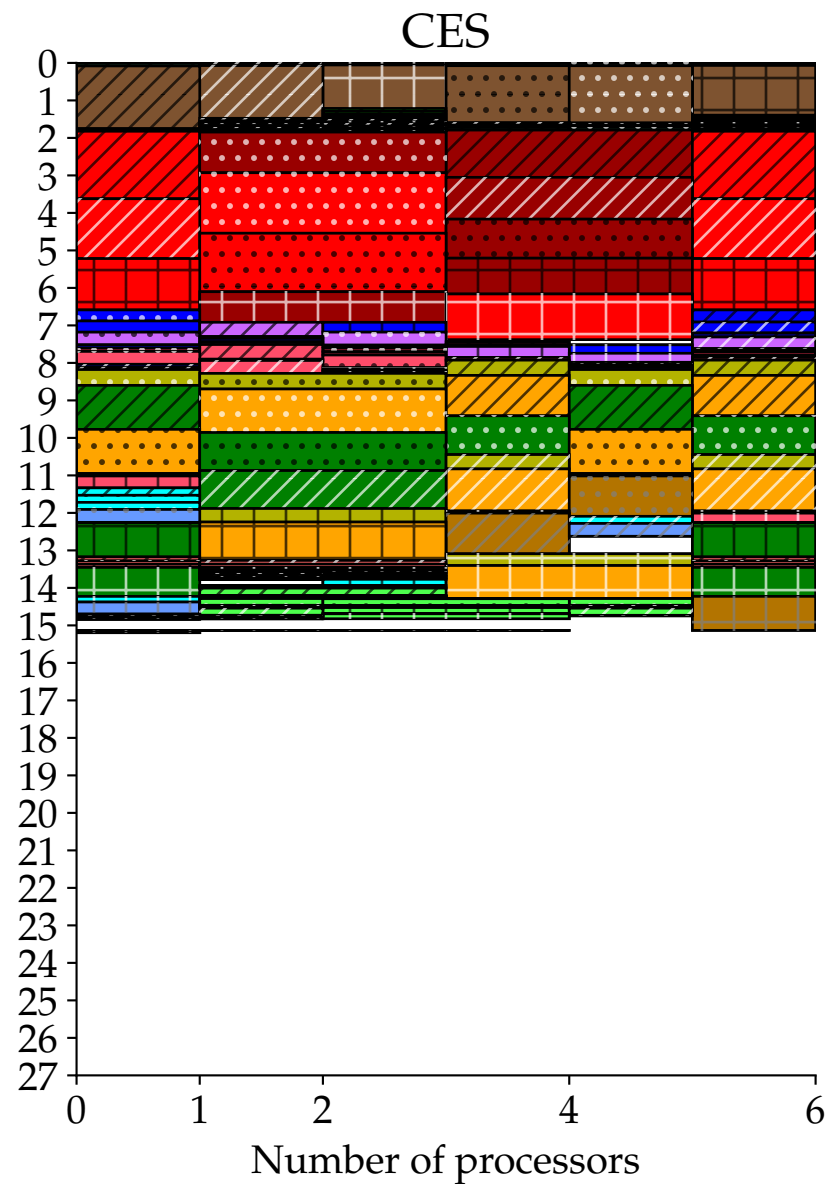

### 3 Samples with 12 processors

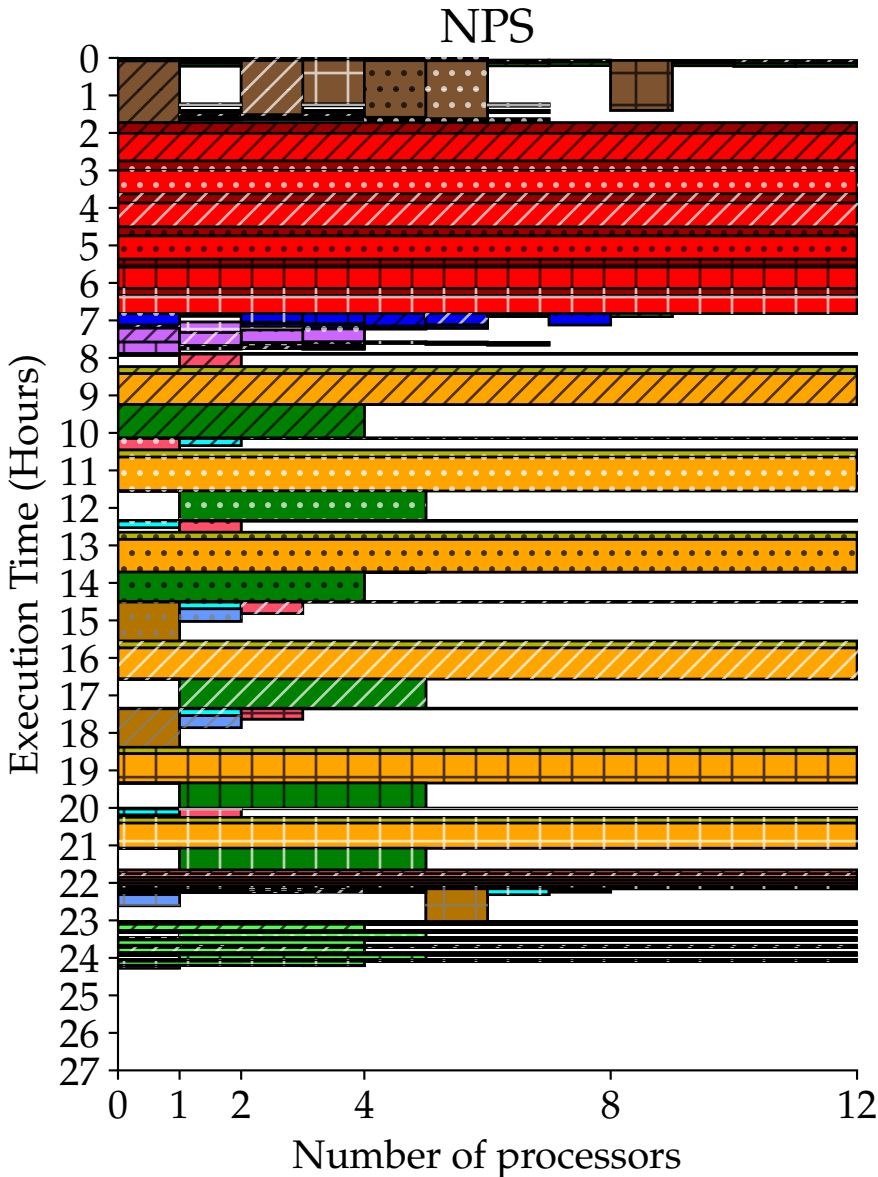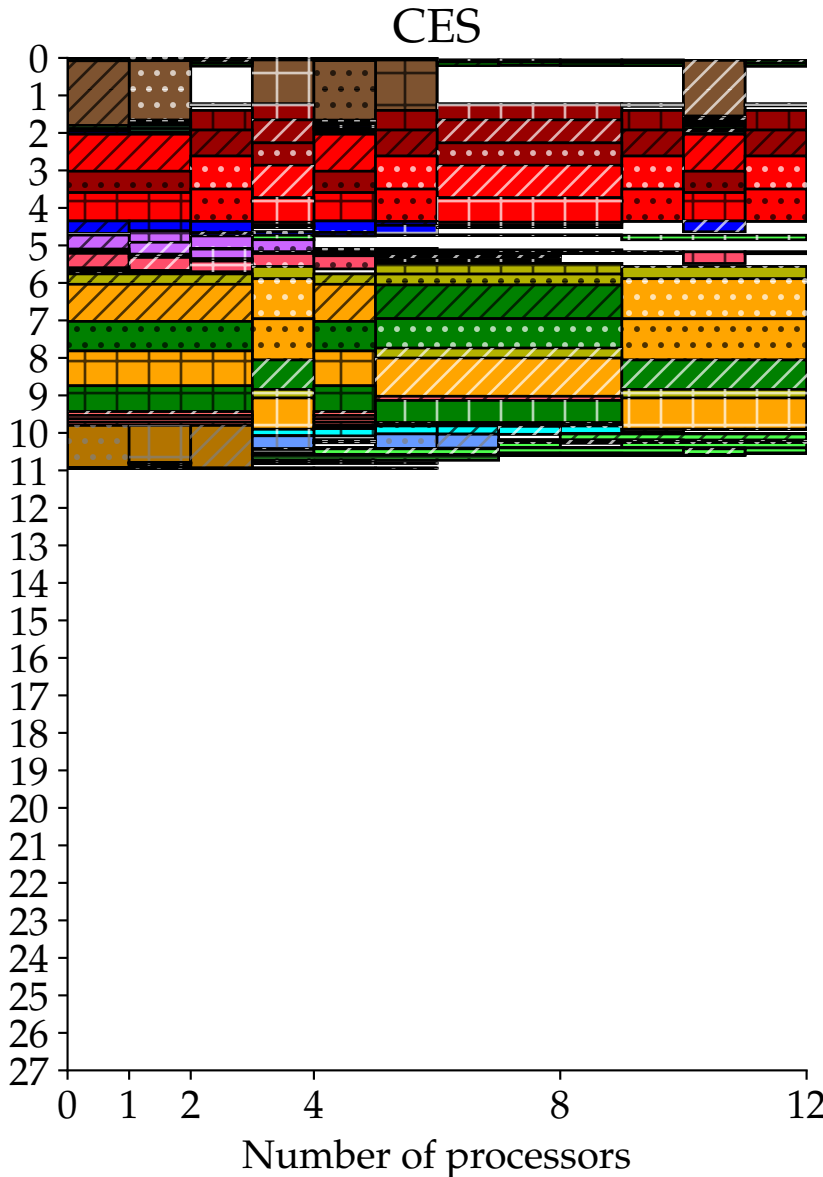

# 3 Samples with 24 processors

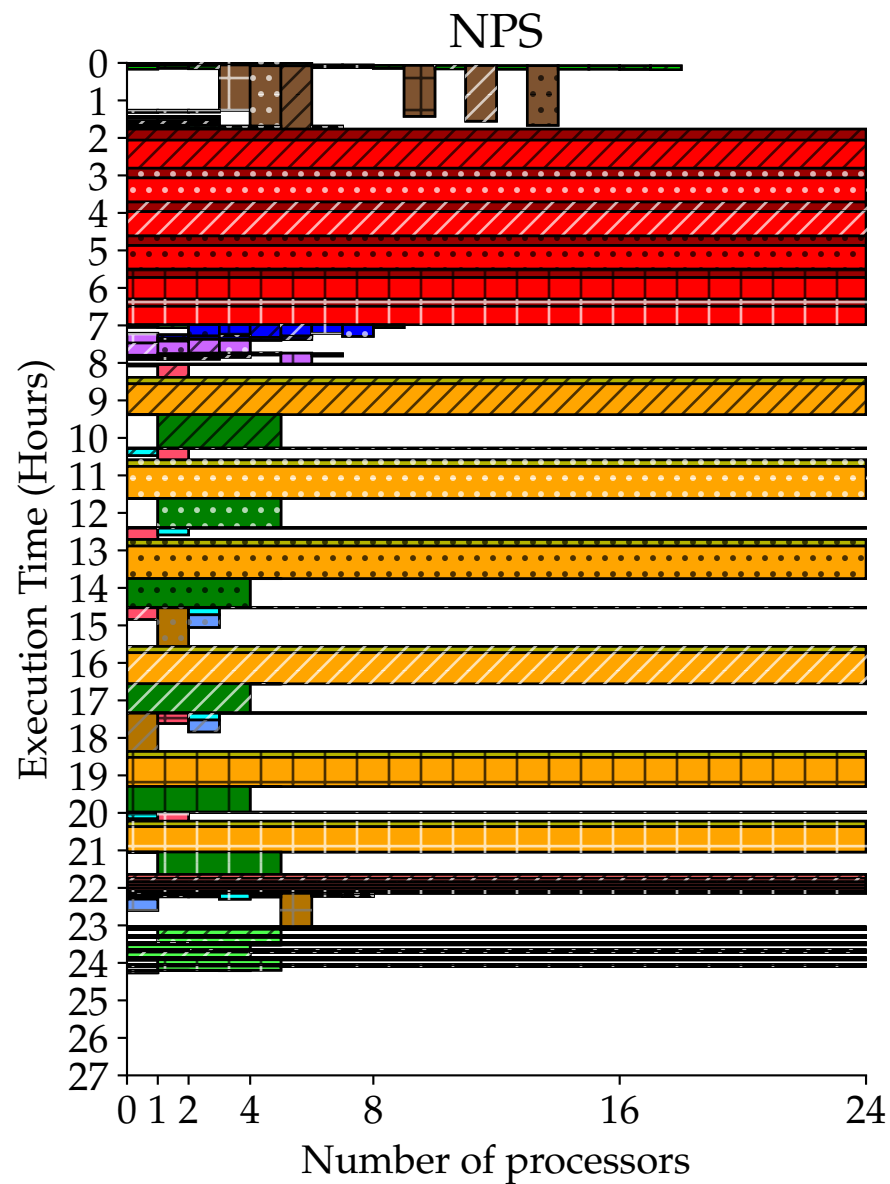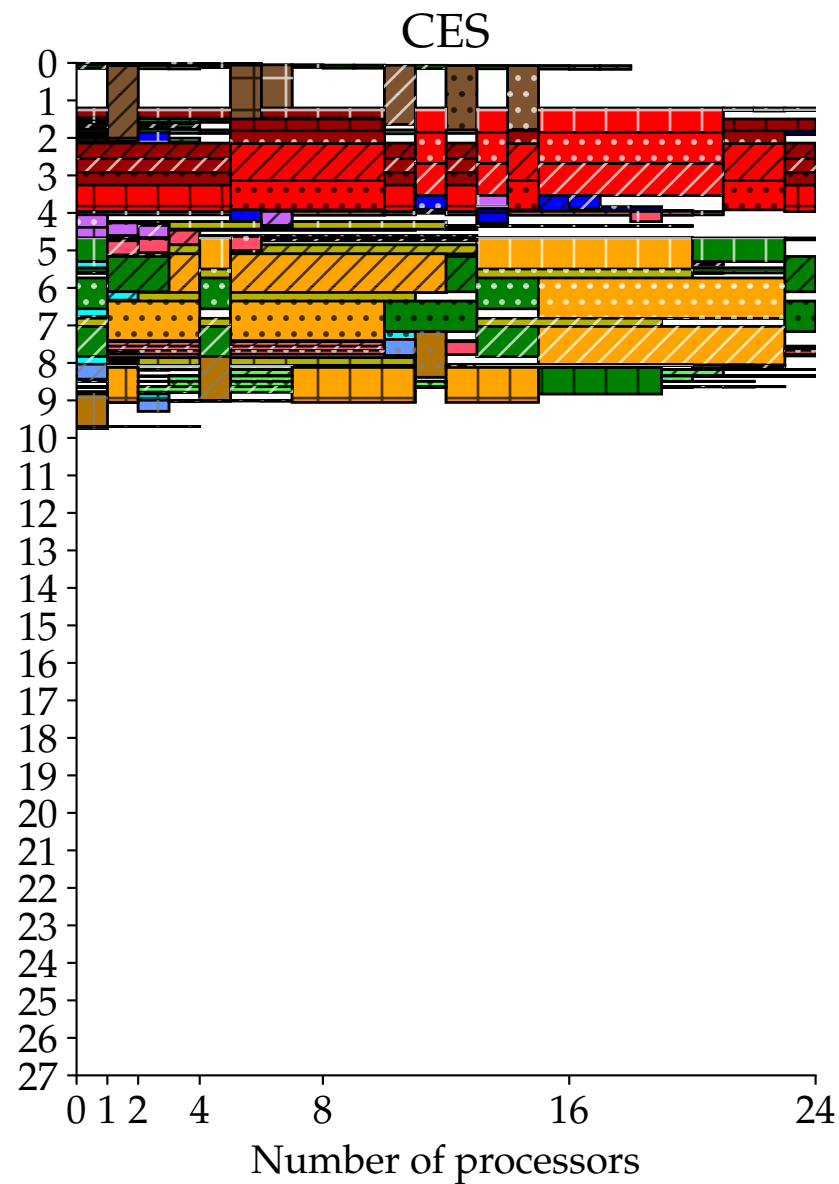

Supplement: Supplementary file 2 — Additional file 2: Figure 1. Representation of all executions (6, 12 and 24 processors) on all 3 samples based on processors usage along time for both NPS and strategies (Fig. 3 as well). [file 12859_2020_3780_MOESM2_ESM.pdf]

# 3 Samples with 6 processors

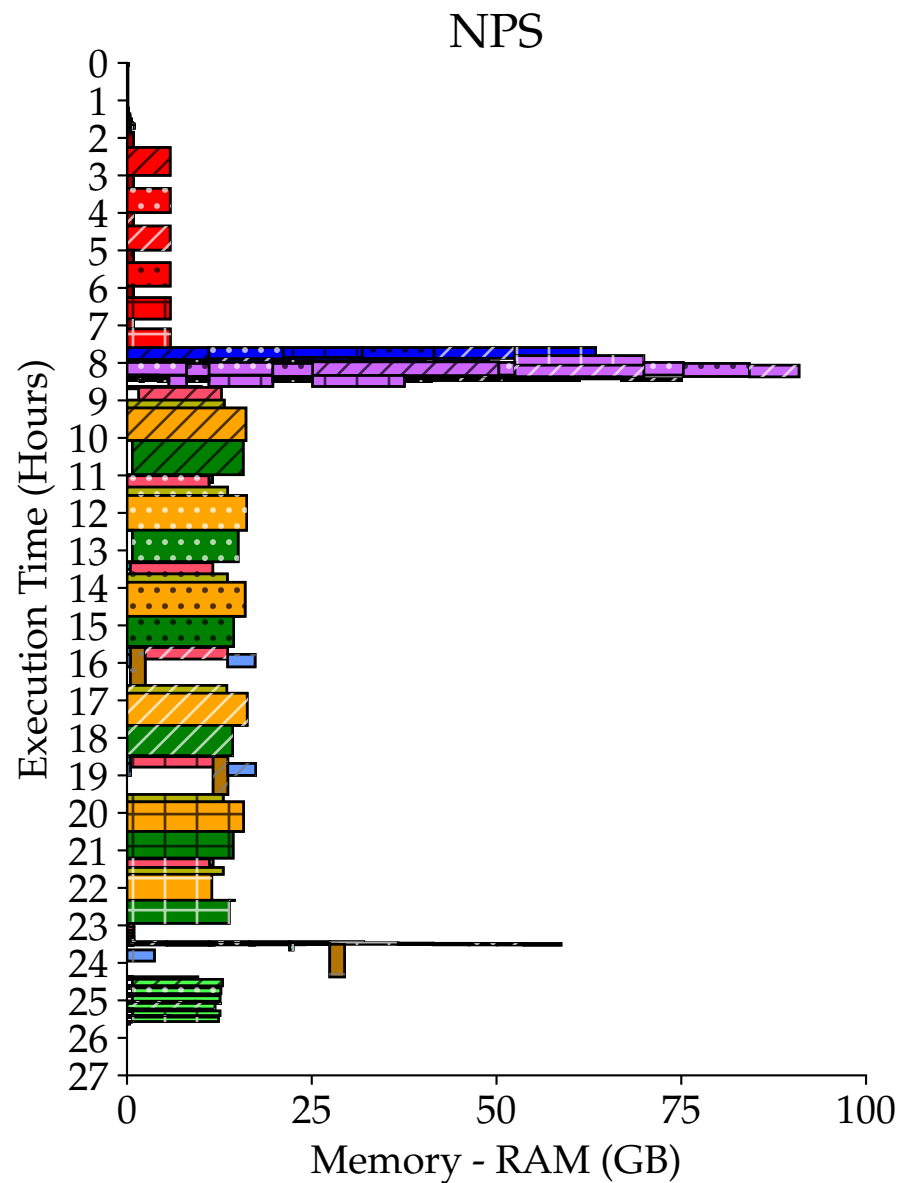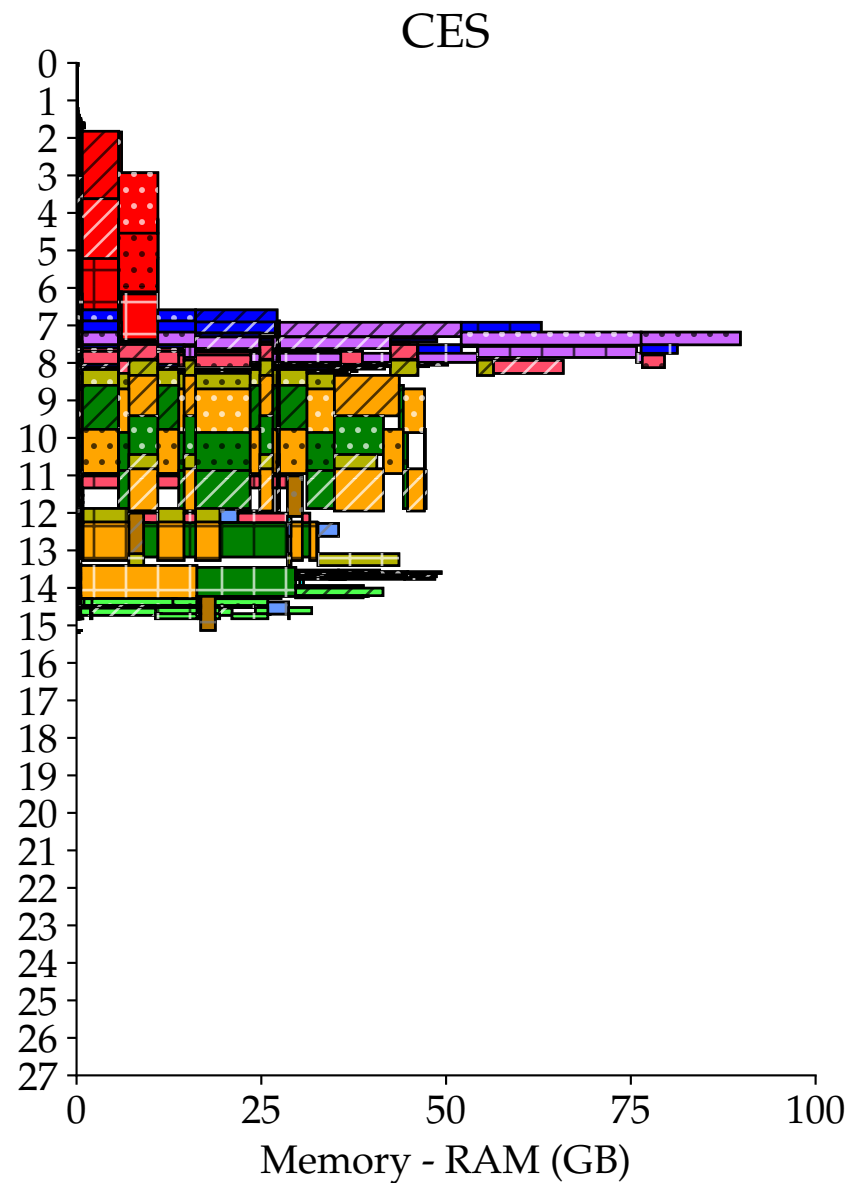

# 3 Samples with 12 processors

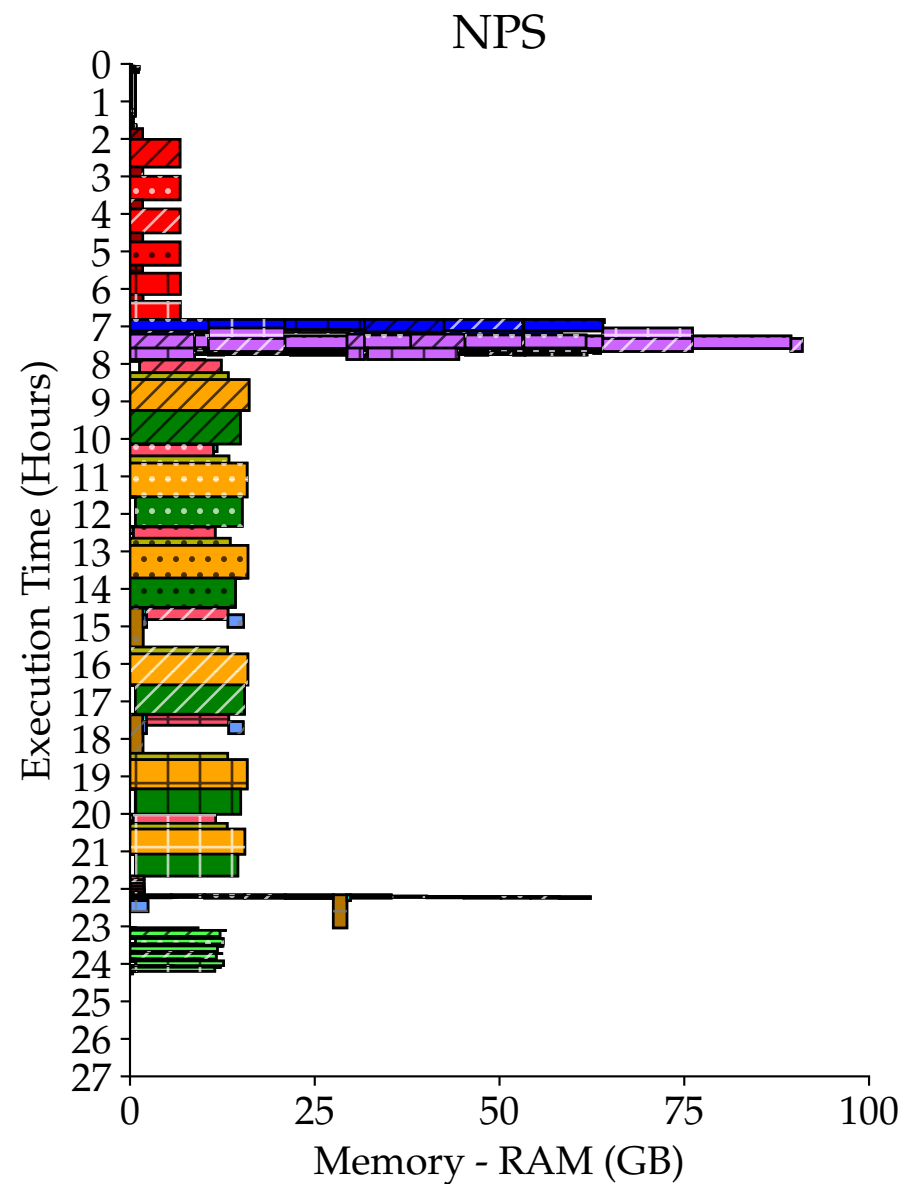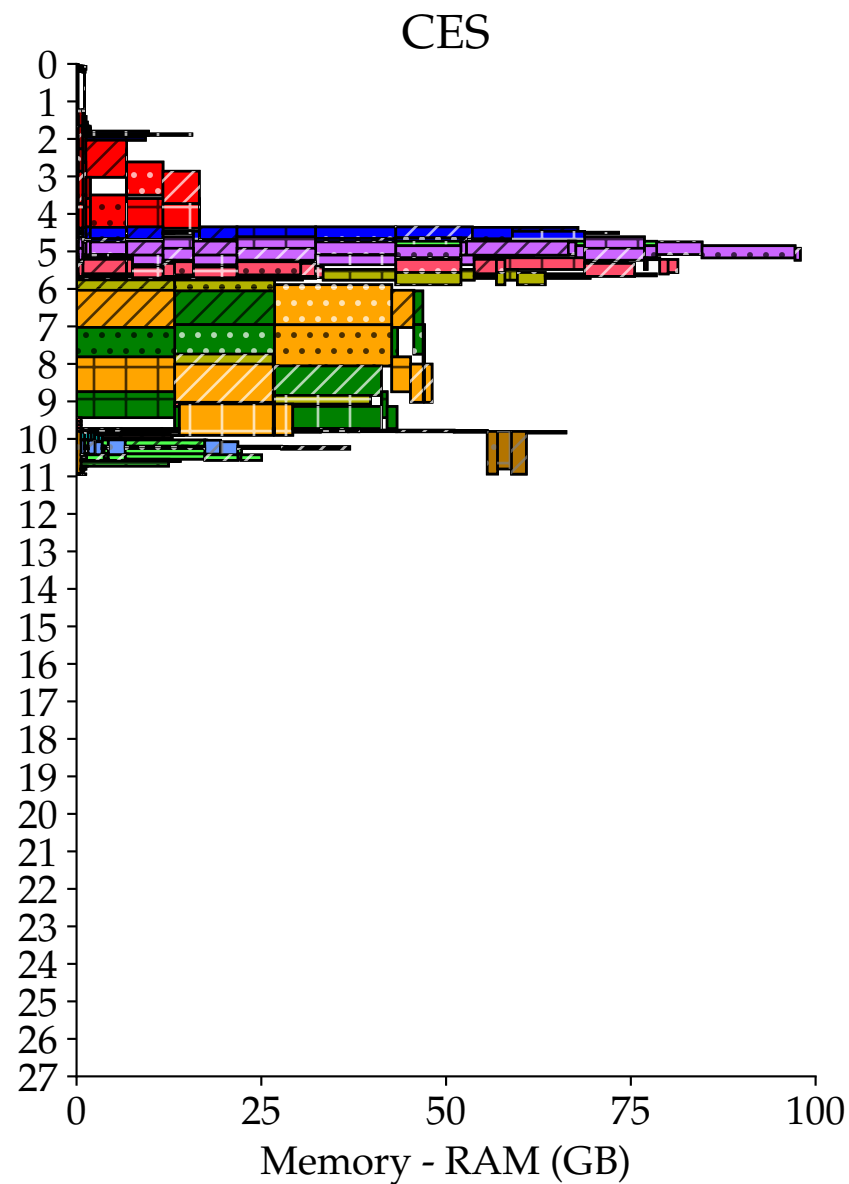

# 3 Samples with 24 processors

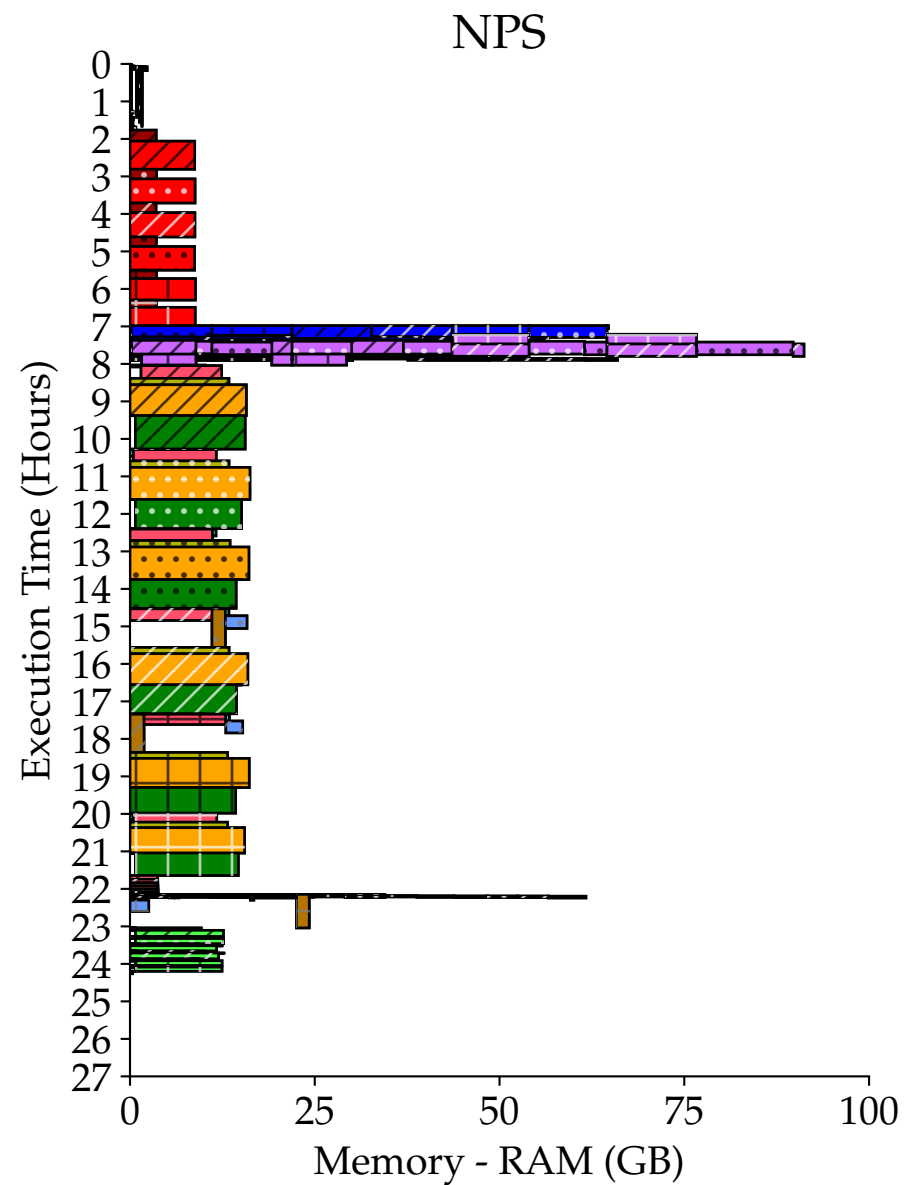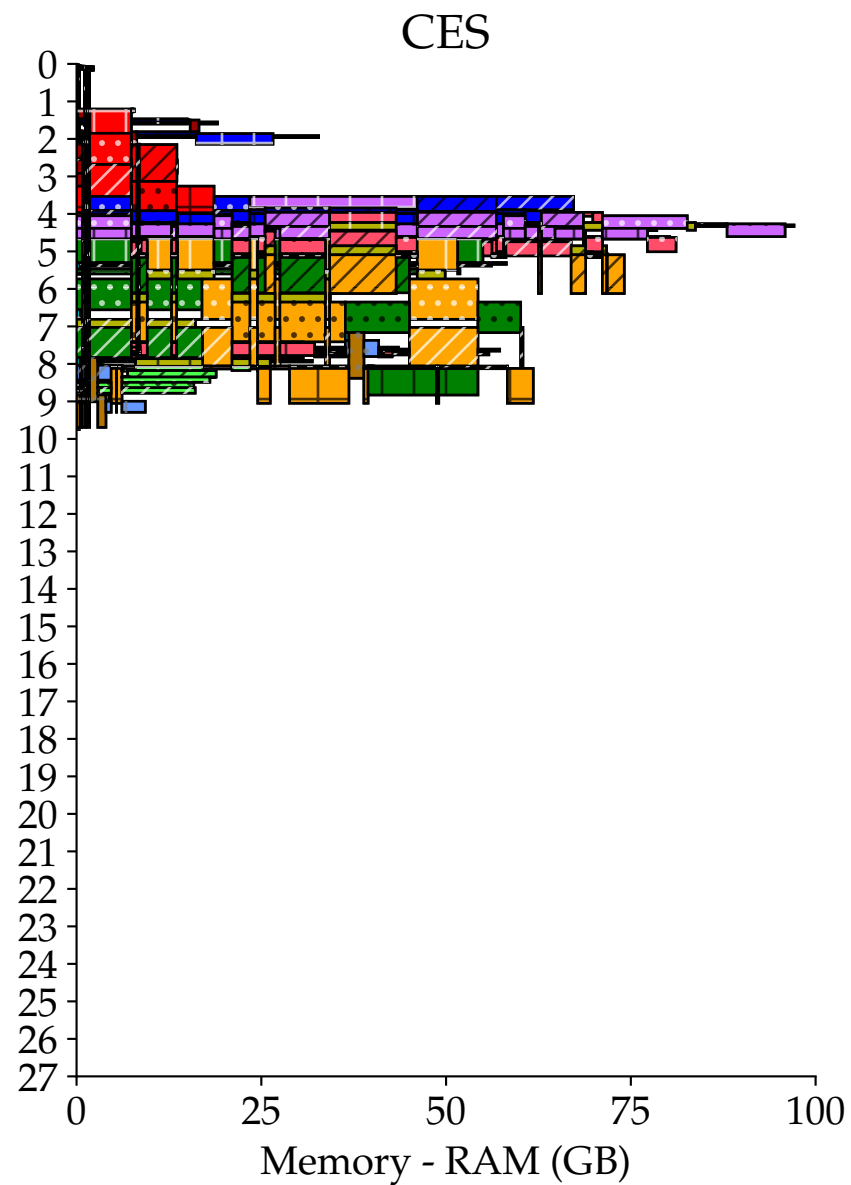

Supplement: Supplementary file 3 — Additional file 3. Figure 2. Representation of all executions (6, 12 and 24 processors) on all 3 samples based on memory usage along time for both NPS and strategies (Fig. 4 as well). [file 12859_2020_3780_MOESM3_ESM.pdf]
