# Supplementary material for: Impact of concurrency on the performance of a whole exome sequencing pipeline
Source: BMC Bioinformatics. 2021 Feb 9;22:60. doi: 10.1186/s12859-020-03780-3 (PMC7874478; doi:10.1186/s12859-020-03780-3)
Supplement: Supplementary file 4 — Additional file 4. Figure 3. Representation of all executions (4, 8 and 16 processors) on 2 samples based on processors usage along time for both NPS and strategies (all possible 2-samples combinations are reported). [file 12859_2020_3780_MOESM4_ESM.pdf]

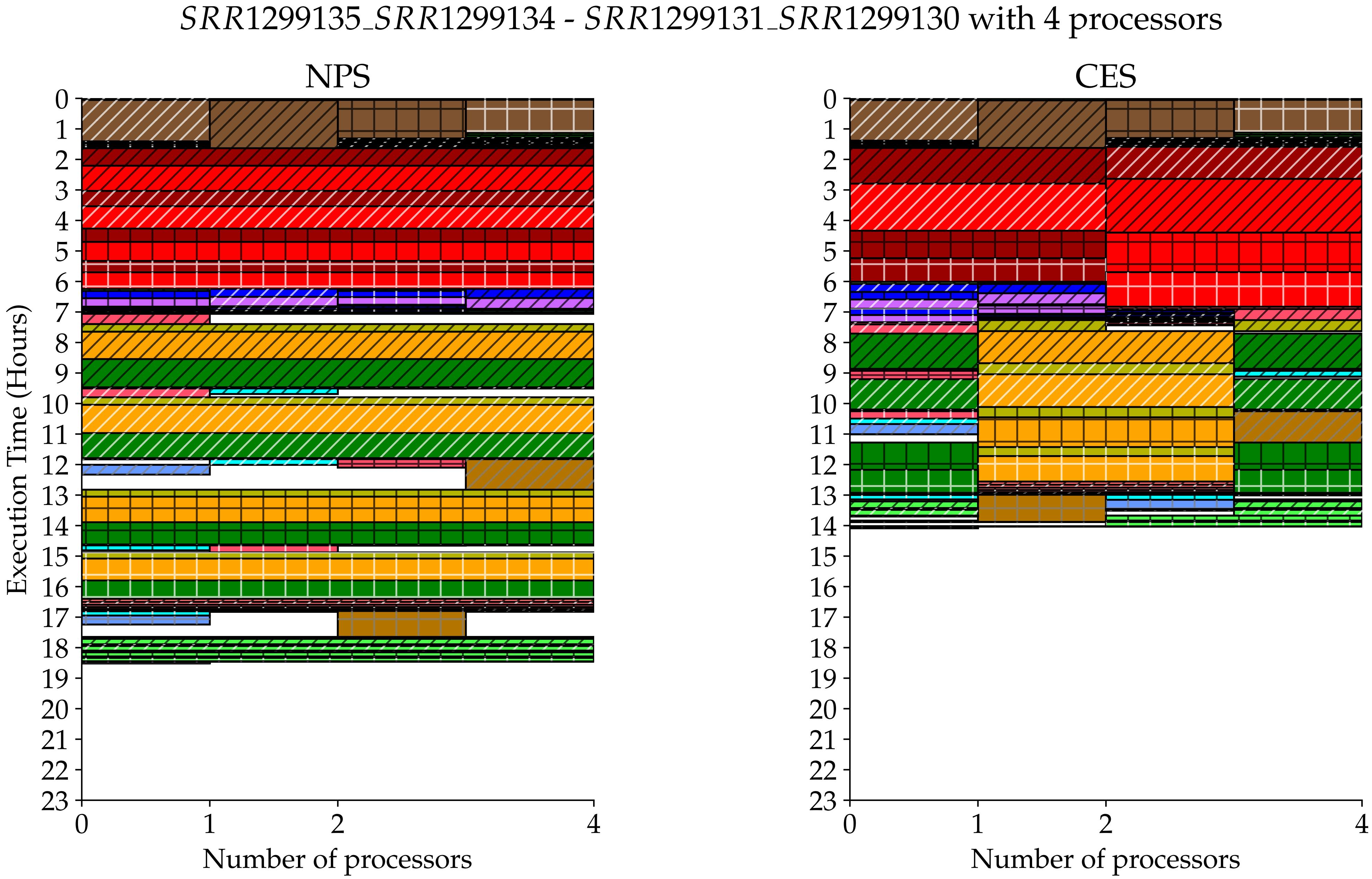
SRR1299135_SRR1299134 - SRR1299131_SRR1299130 with 4 processors
NPS
CES
Execution Time (Hours)
Number of processors
0
1
2
4
0
1
2
3
4
5
6
7
8
9
10
11
12
13
14
15
16
17
18
19
20
21
22
23

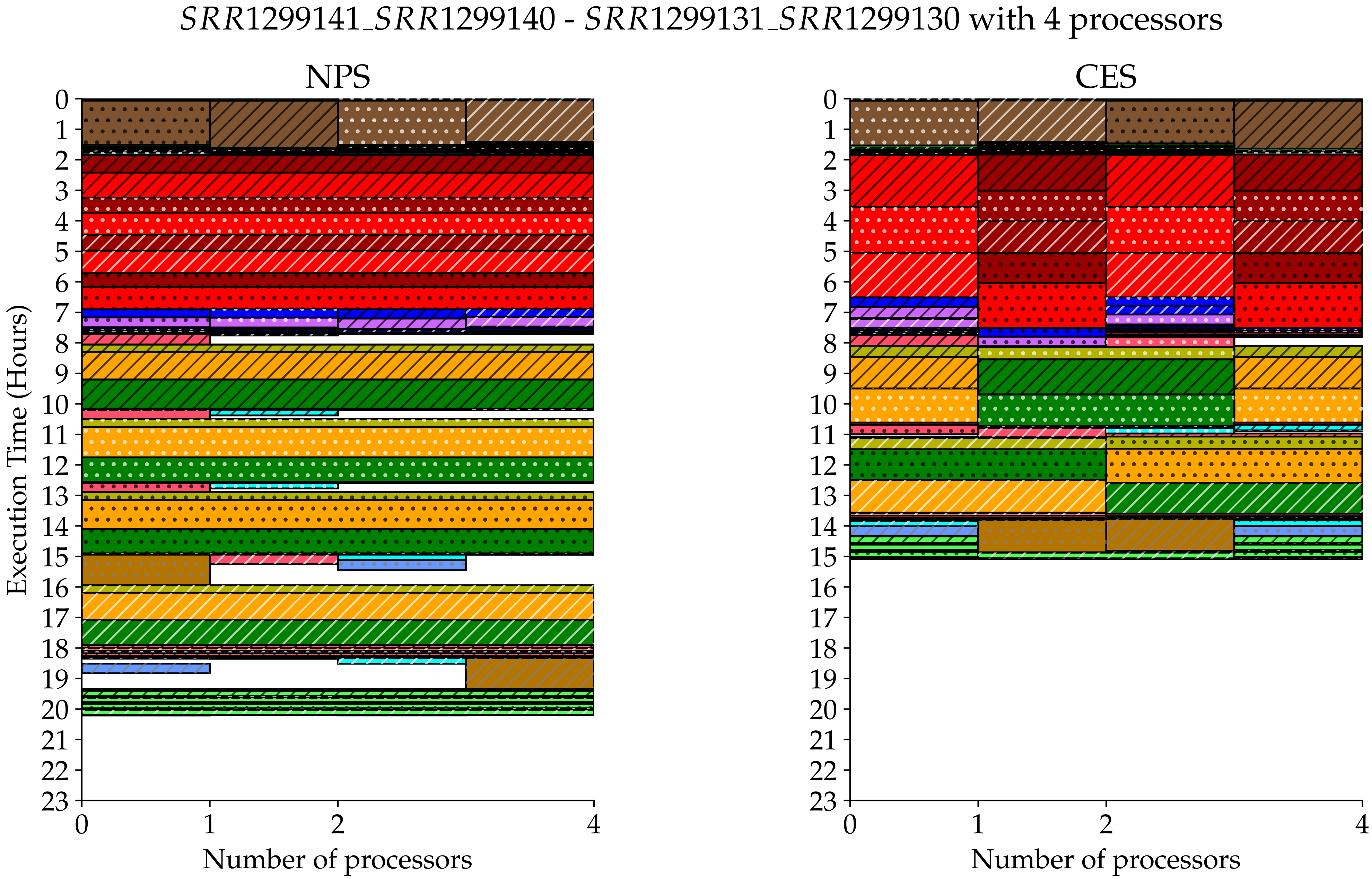

SRR1299141_SRR1299140 - SRR1299131_SRR1299130 with 4 processors
NPS
CES
Execution Time (Hours)
Number of processors
Number of processors

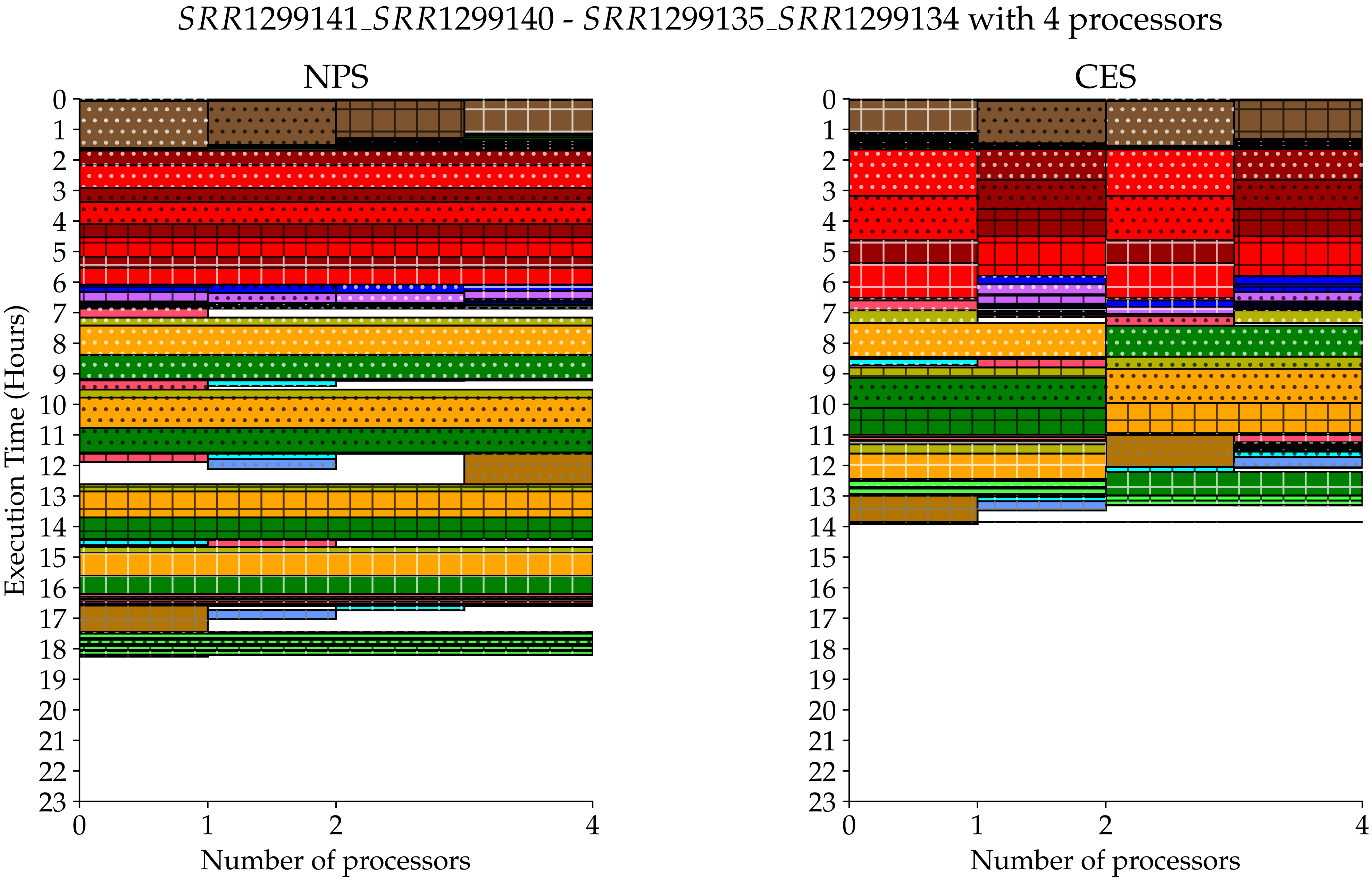
$SRR1299141\_SRR1299140$ - $SRR1299135\_SRR1299134$ with 4 processors
NPS
CES
Execution Time (Hours)
Number of processors
Number of processors
0
1
2
3
4
5
6
7
8
9
10
11
12
13
14
15
16
17
18
19
20
21
22
23
0
1
2
4

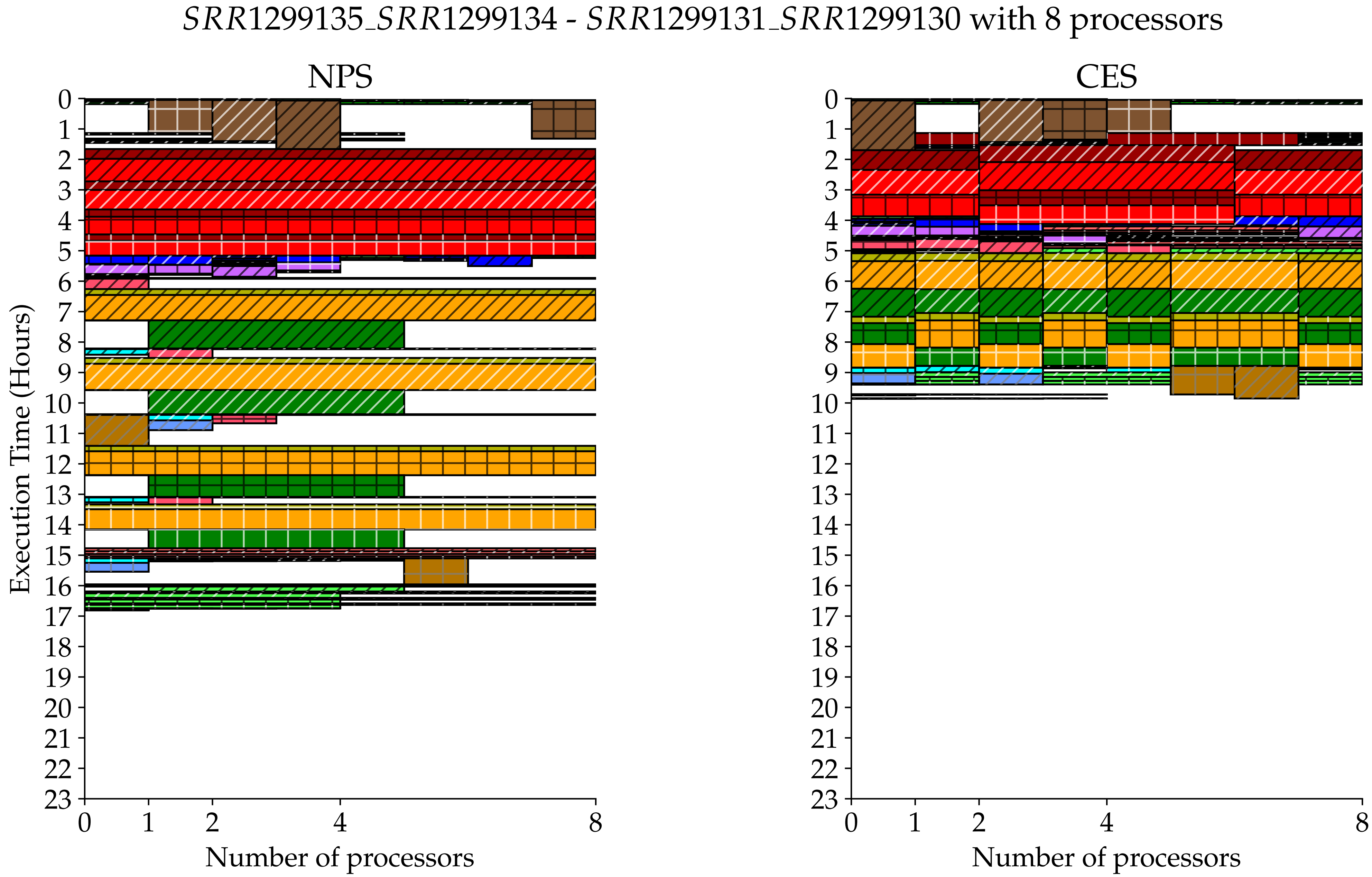

$SRR1299135\_SRR1299134$ - $SRR1299131\_SRR1299130$ with 8 processors
NPS
CES
Execution Time (Hours)
0
1
2
3
4
5
6
7
8
9
10
11
12
13
14
15
16
17
18
19
20
21
22
23
0
1
2
4
8
Number of processors
Number of processors

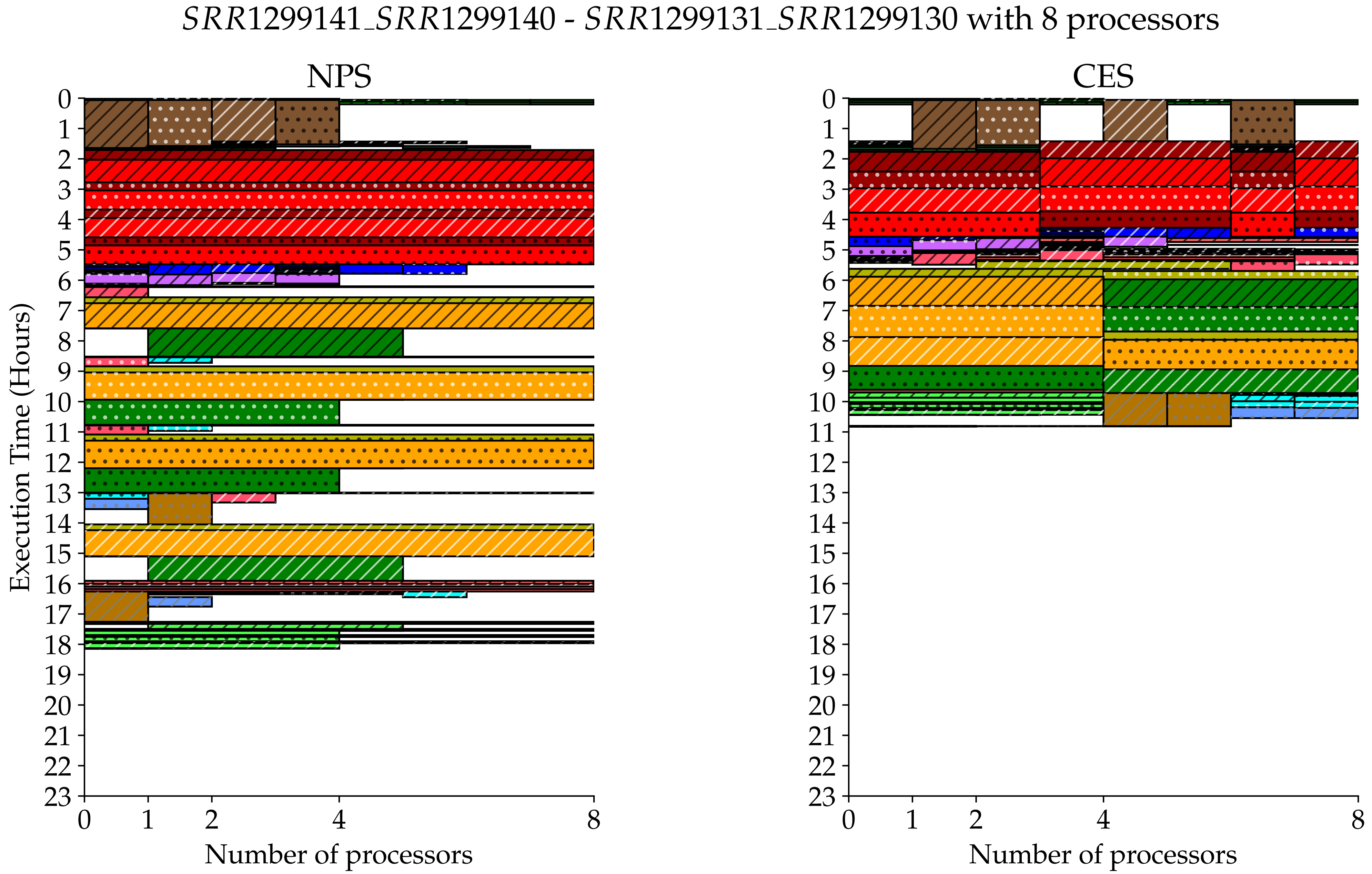
$SRR1299141\_SRR1299140$ - $SRR1299131\_SRR1299130$ with 8 processors
NPS
CES
Execution Time (Hours)
Number of processors
Number of processors

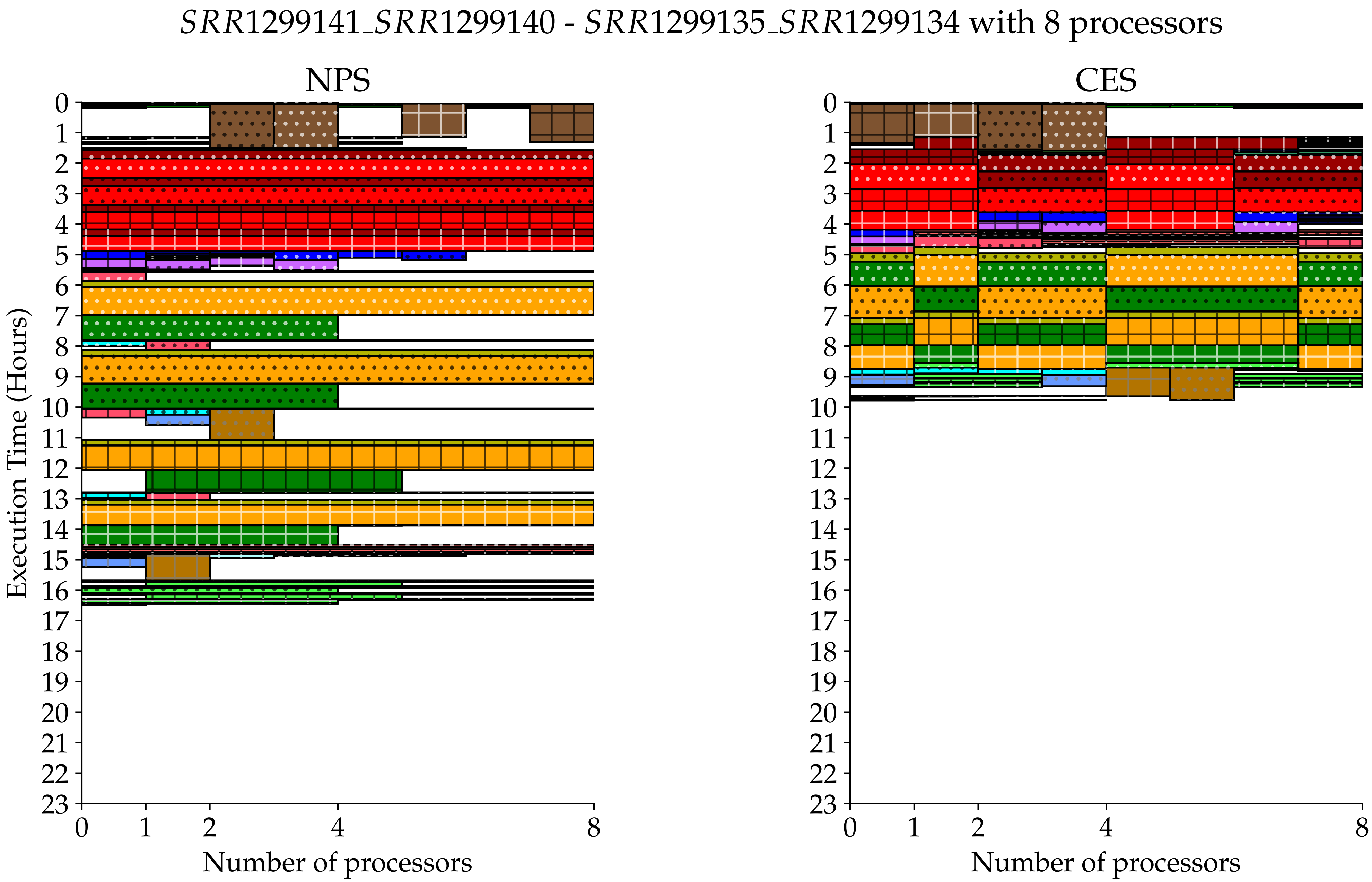
$SRR1299141\_SRR1299140$ - $SRR1299135\_SRR1299134$ with 8 processors
NPS
CES
Execution Time (Hours)
0
1
2
3
4
5
6
7
8
9
10
11
12
13
14
15
16
17
18
19
20
21
22
23
Number of processors
0
1
2
4
8

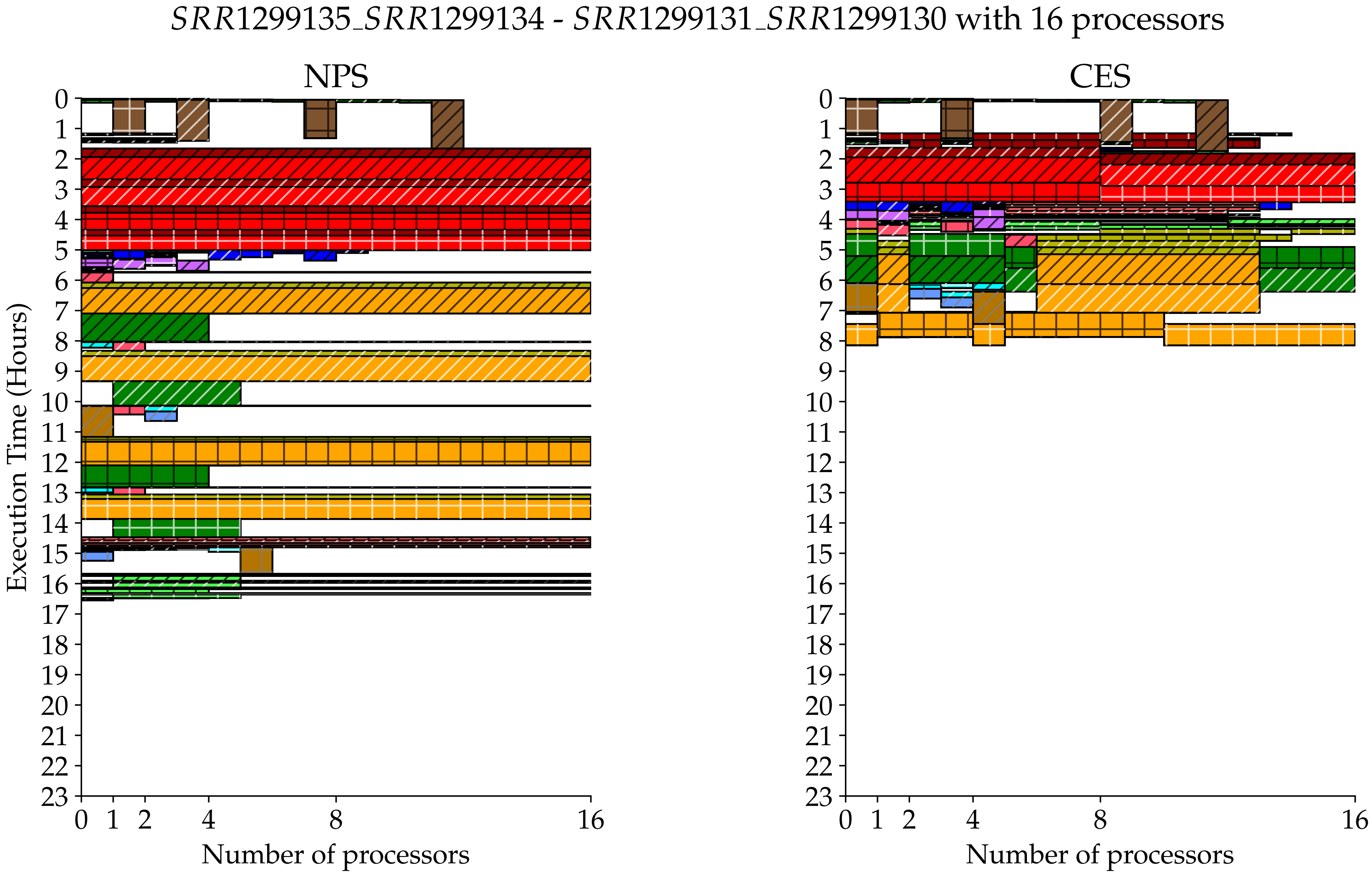

$SRR1299135\_SRR1299134$ - $SRR1299131\_SRR1299130$ with 16 processors
NPS
CES
Execution Time (Hours)
Number of processors
Number of processors
0 1 2 4 8 16
0 1 2 3 4 5 6 7 8 9 10 11 12 13 14 15 16 17 18 19 20 21 22 23

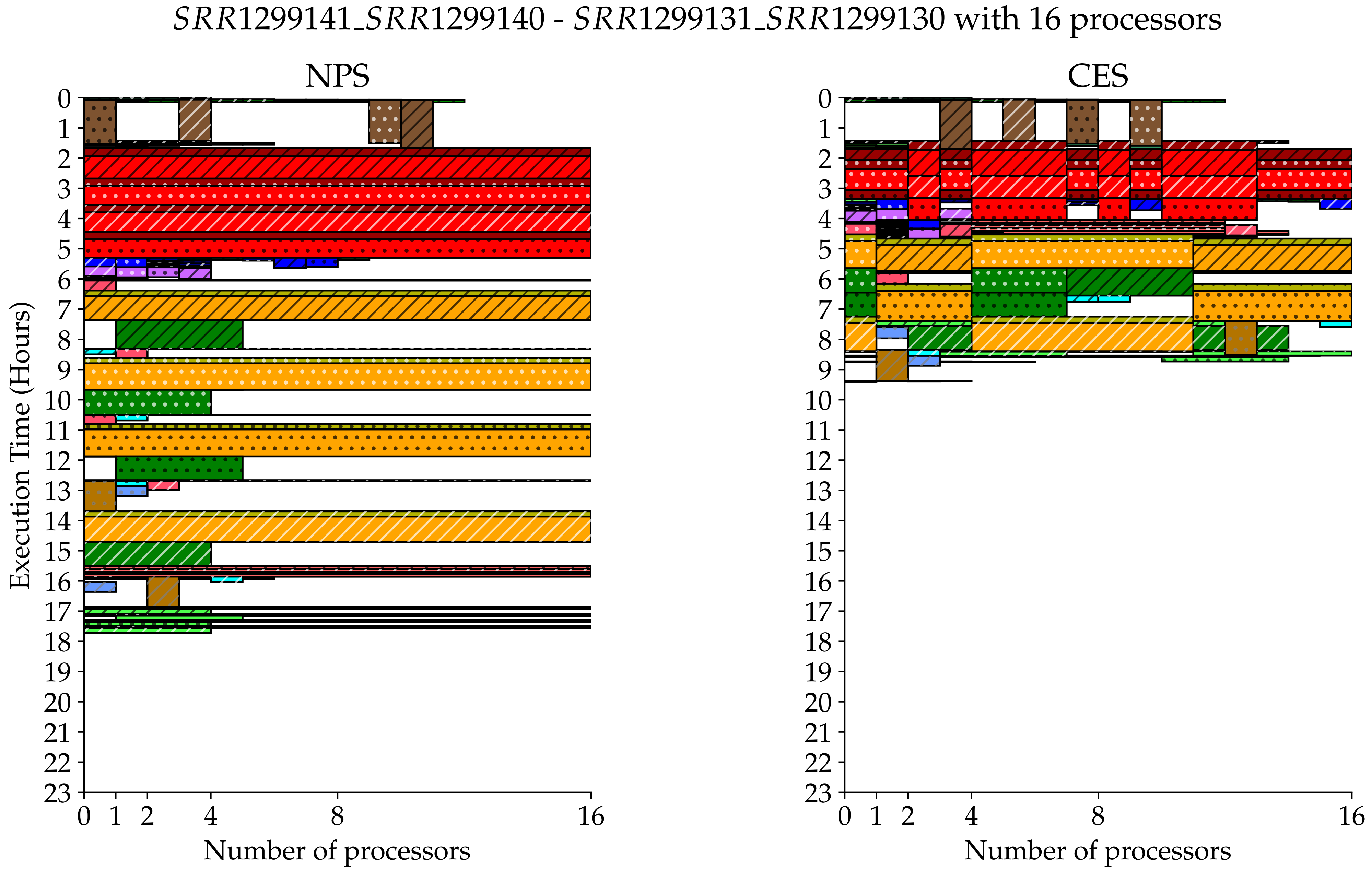

$SRR1299141\_SRR1299140$ - $SRR1299131\_SRR1299130$ with 16 processors
NPS
CES
Execution Time (Hours)
Number of processors
Number of processors

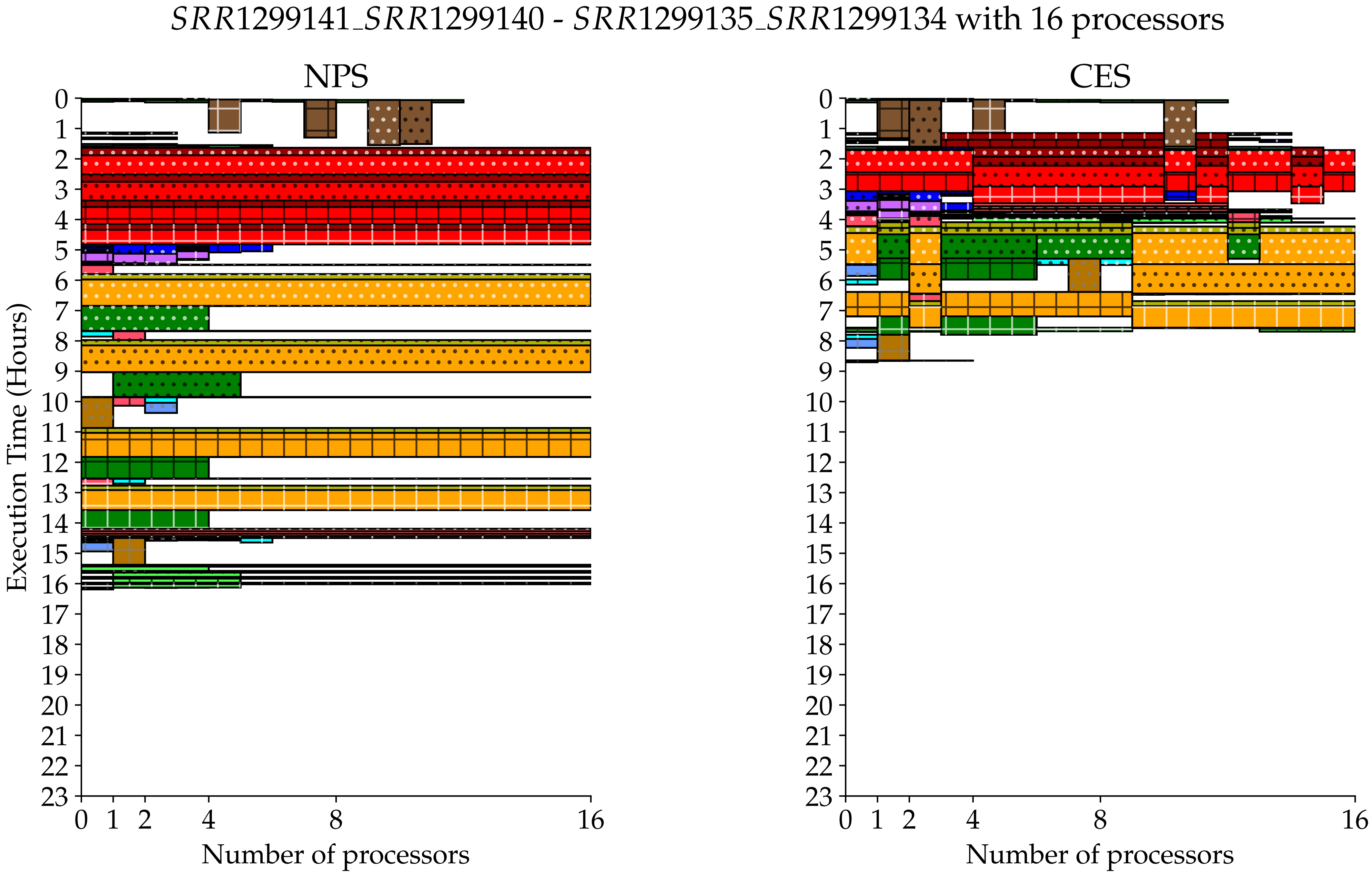

$SRR1299141\_SRR1299140$ - $SRR1299135\_SRR1299134$ with 16 processors
NPS
CES
Execution Time (Hours)
0
1
2
3
4
5
6
7
8
9
10
11
12
13
14
15
16
17
18
19
20
21
22
23
0
1
2
4
8
16
Number of processors
