# Supplementary material for: Impact of concurrency on the performance of a whole exome sequencing pipeline
Source: BMC Bioinformatics. 2021 Feb 9;22:60. doi: 10.1186/s12859-020-03780-3 (PMC7874478; doi:10.1186/s12859-020-03780-3)
Supplement: Supplementary file 5 — Additional file 5. Figure 4. Representation of all executions (4, 8 and 16 processors) on 2 samples based on memory usage along time for both NPS and strategies (all possible 2-samples combinations are reported). [file 12859_2020_3780_MOESM5_ESM.pdf]

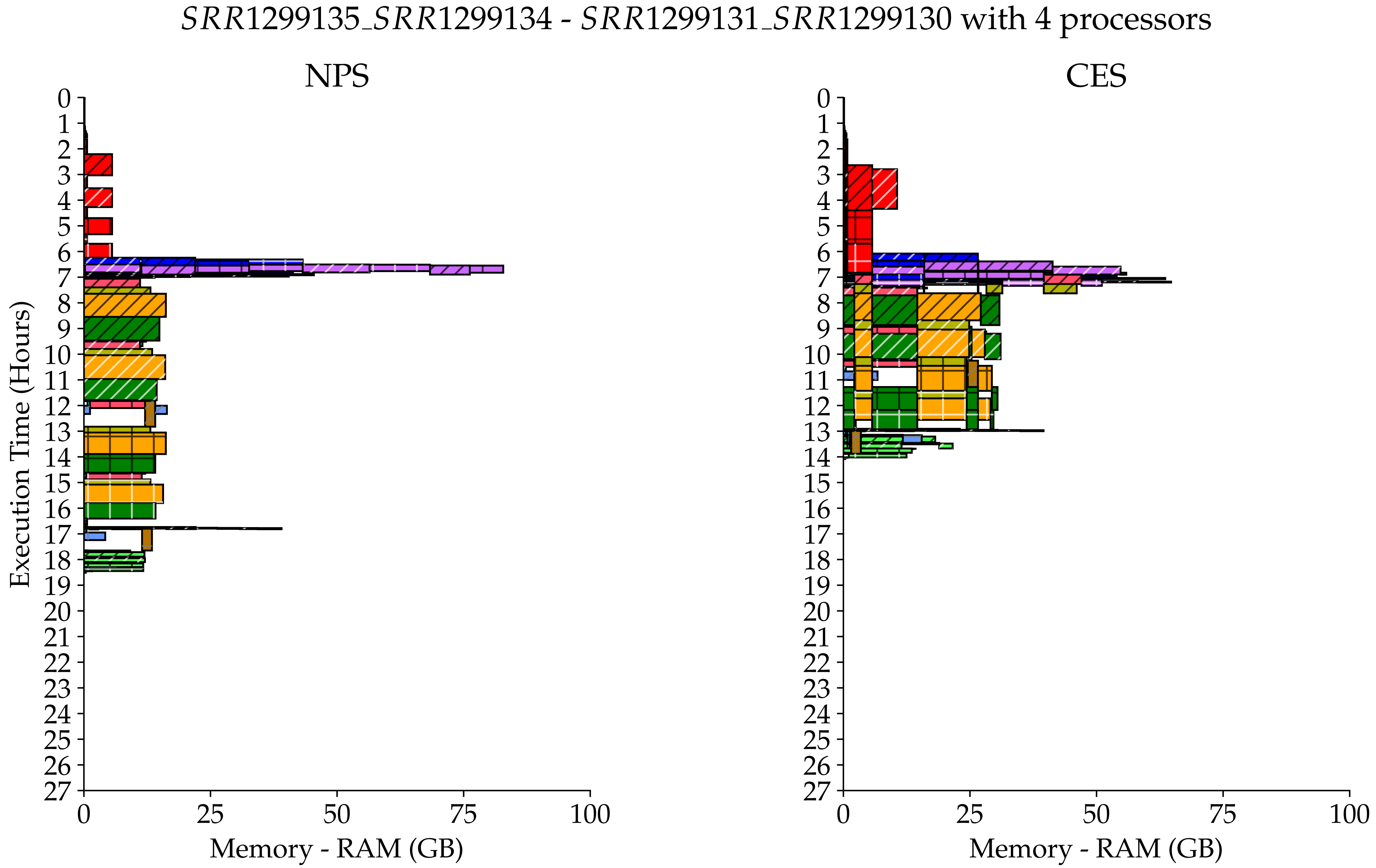

$SRR1299135\_SRR1299134$ - $SRR1299131\_SRR1299130$ with 4 processors
NPS
CES
Execution Time (Hours)
Memory - RAM (GB)
0
1
2
3
4
5
6
7
8
9
10
11
12
13
14
15
16
17
18
19
20
21
22
23
24
25
26
27
0
25
50
75
100

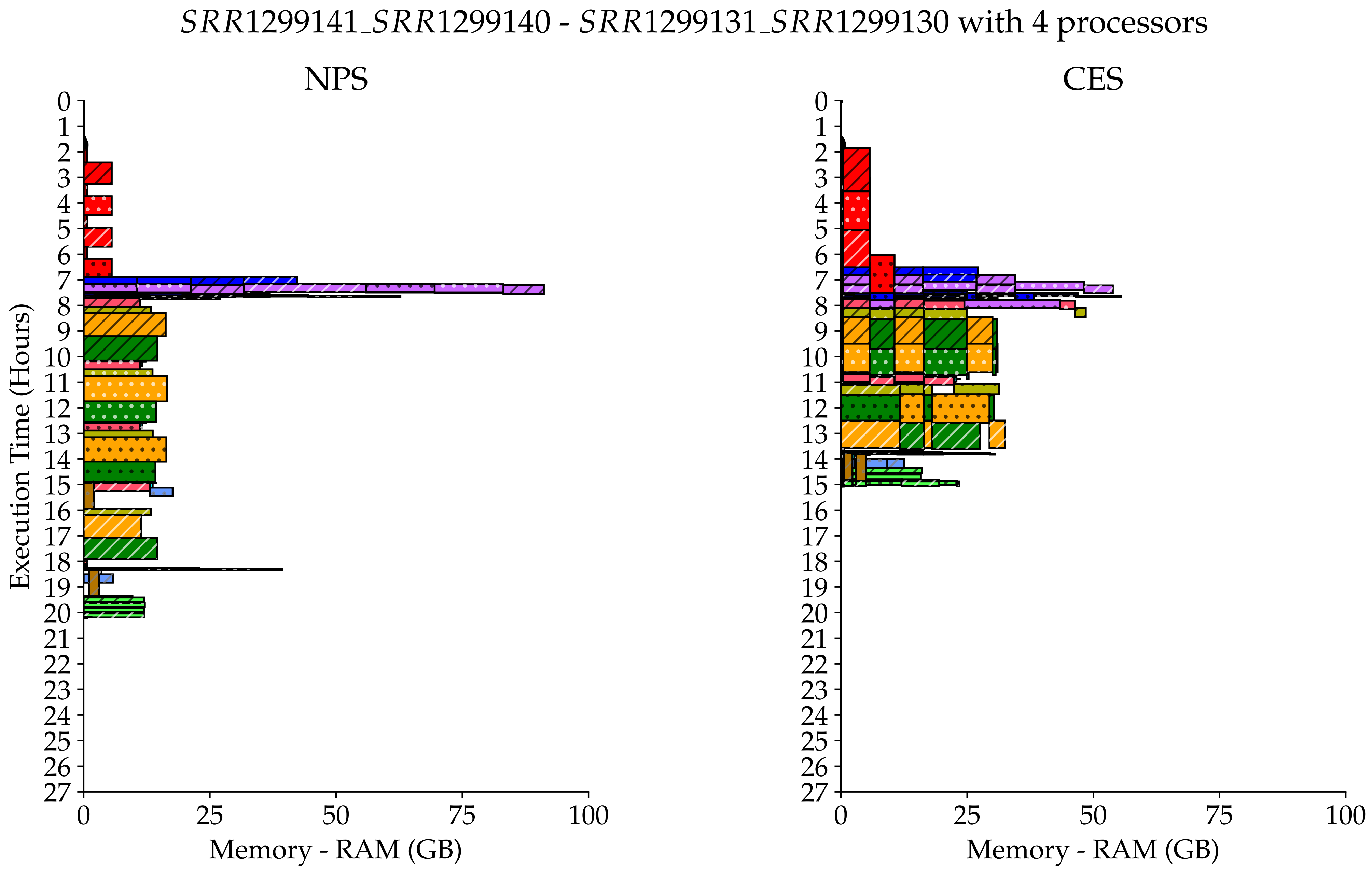

$SRR1299141\_SRR1299140$ - $SRR1299131\_SRR1299130$ with 4 processors
NPS
CES
Execution Time (Hours)
Memory - RAM (GB)
Memory - RAM (GB)

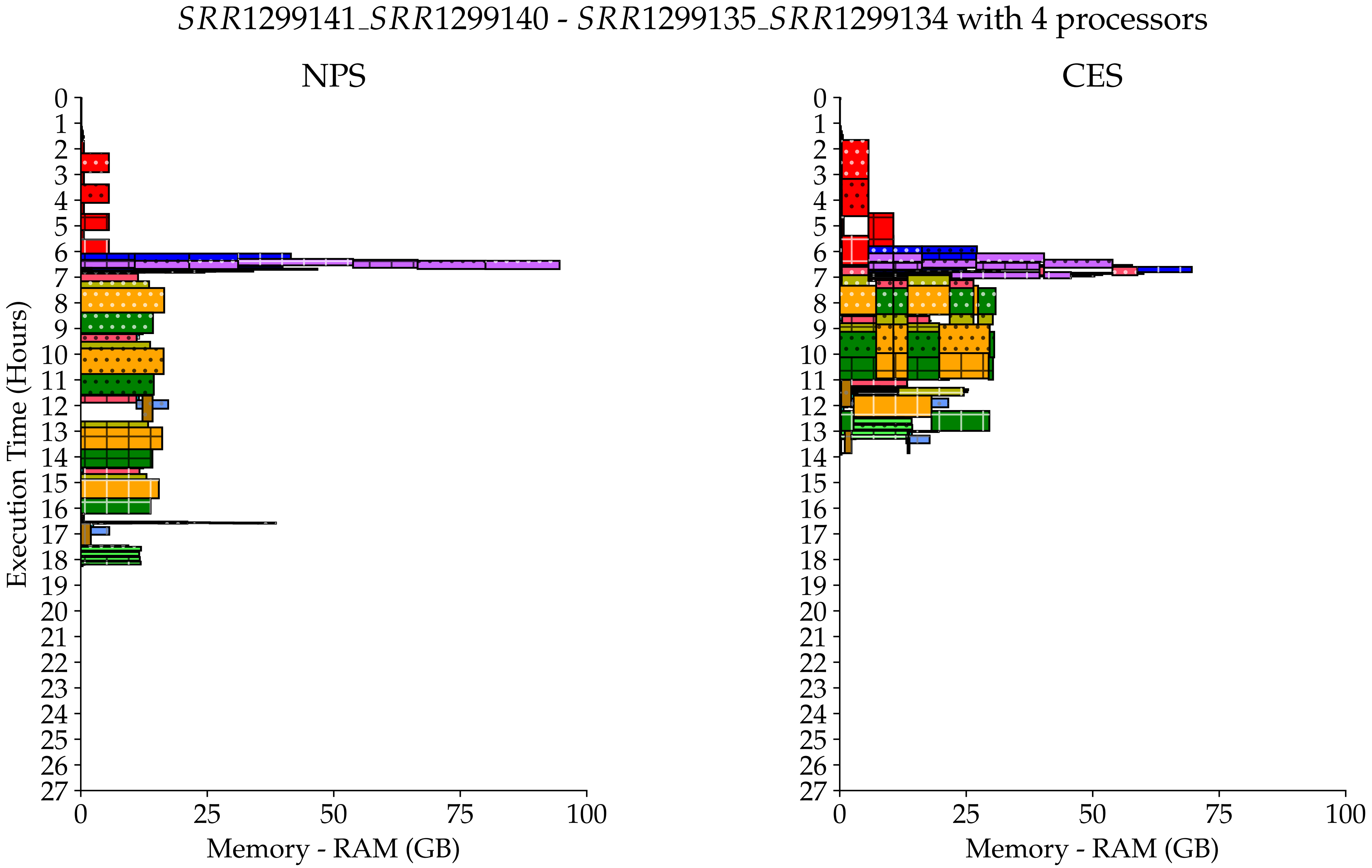

SRR1299141_SRR1299140 - SRR1299135_SRR1299134 with 4 processors
NPS
CES
Execution Time (Hours)
Memory - RAM (GB)
0
25
50
75
100

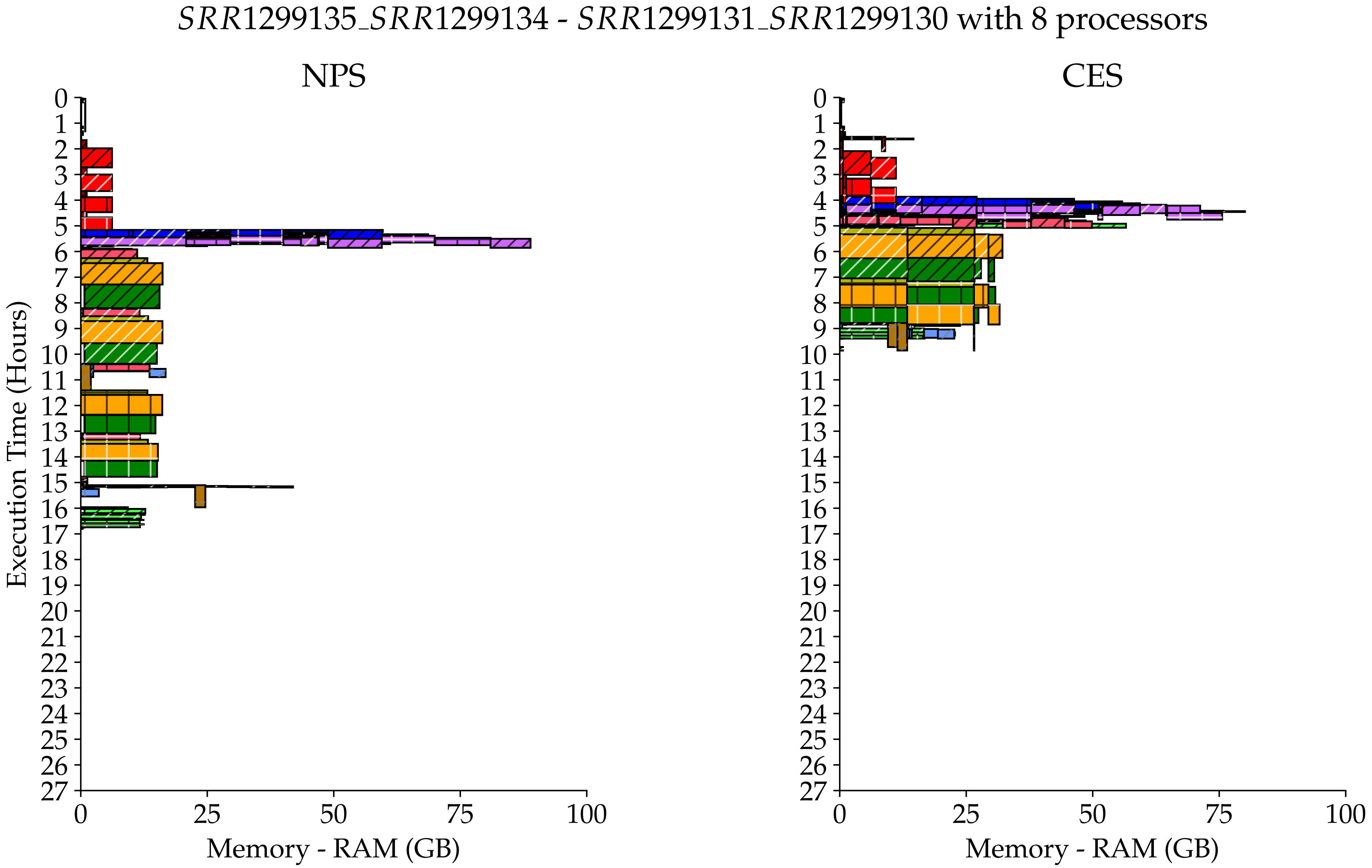
$SRR1299135\_SRR1299134$ - $SRR1299131\_SRR1299130$ with 8 processors
NPS
CES
Execution Time (Hours)
Memory - RAM (GB)
Memory - RAM (GB)
0
1
2
3
4
5
6
7
8
9
10
11
12
13
14
15
16
17
18
19
20
21
22
23
24
25
26
27
0
25
50
75
100

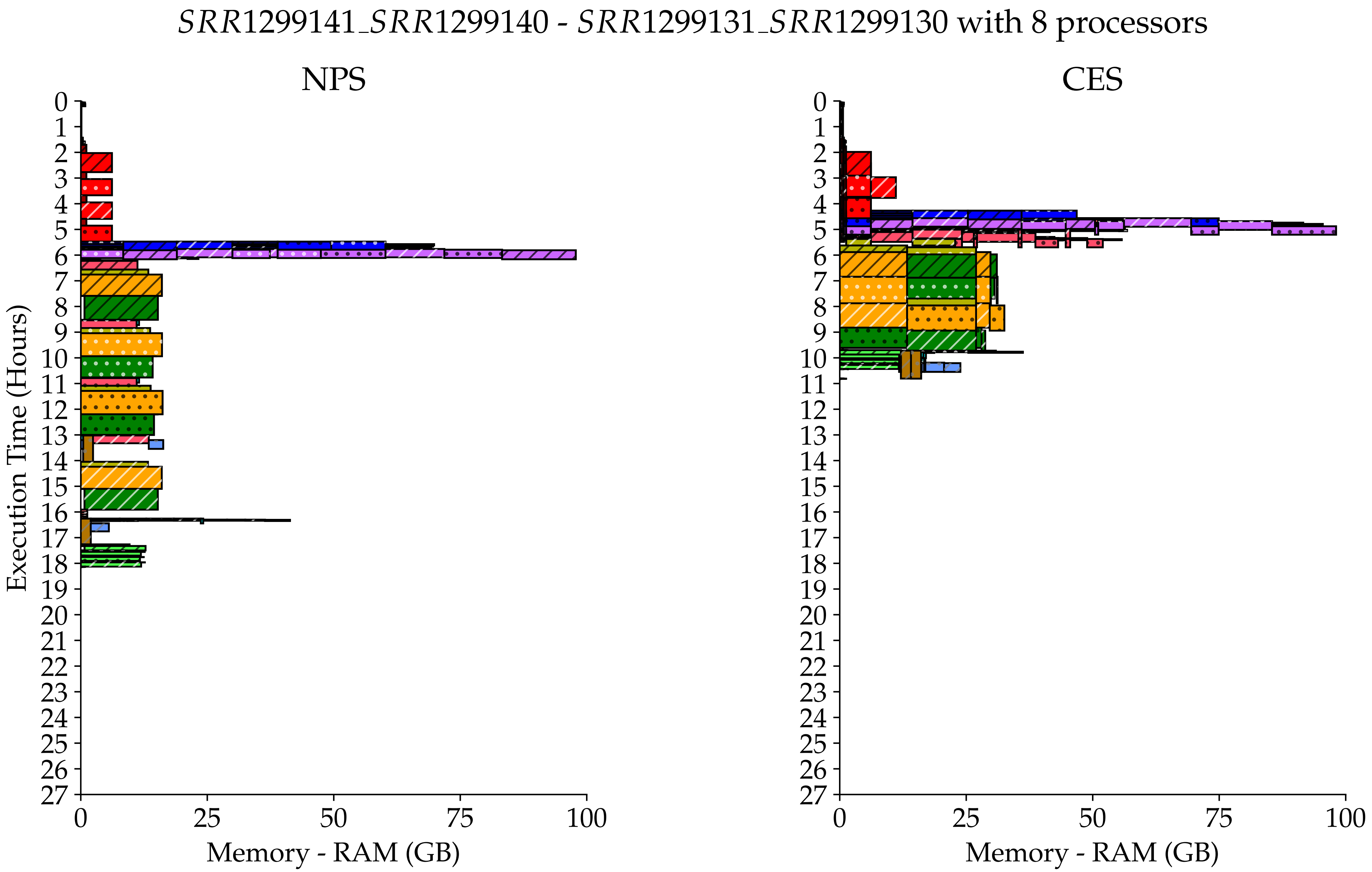

$SRR1299141\_SRR1299140$ - $SRR1299131\_SRR1299130$ with 8 processors
NPS
CES
Execution Time (Hours)
Memory - RAM (GB)
0
25
50
75
100

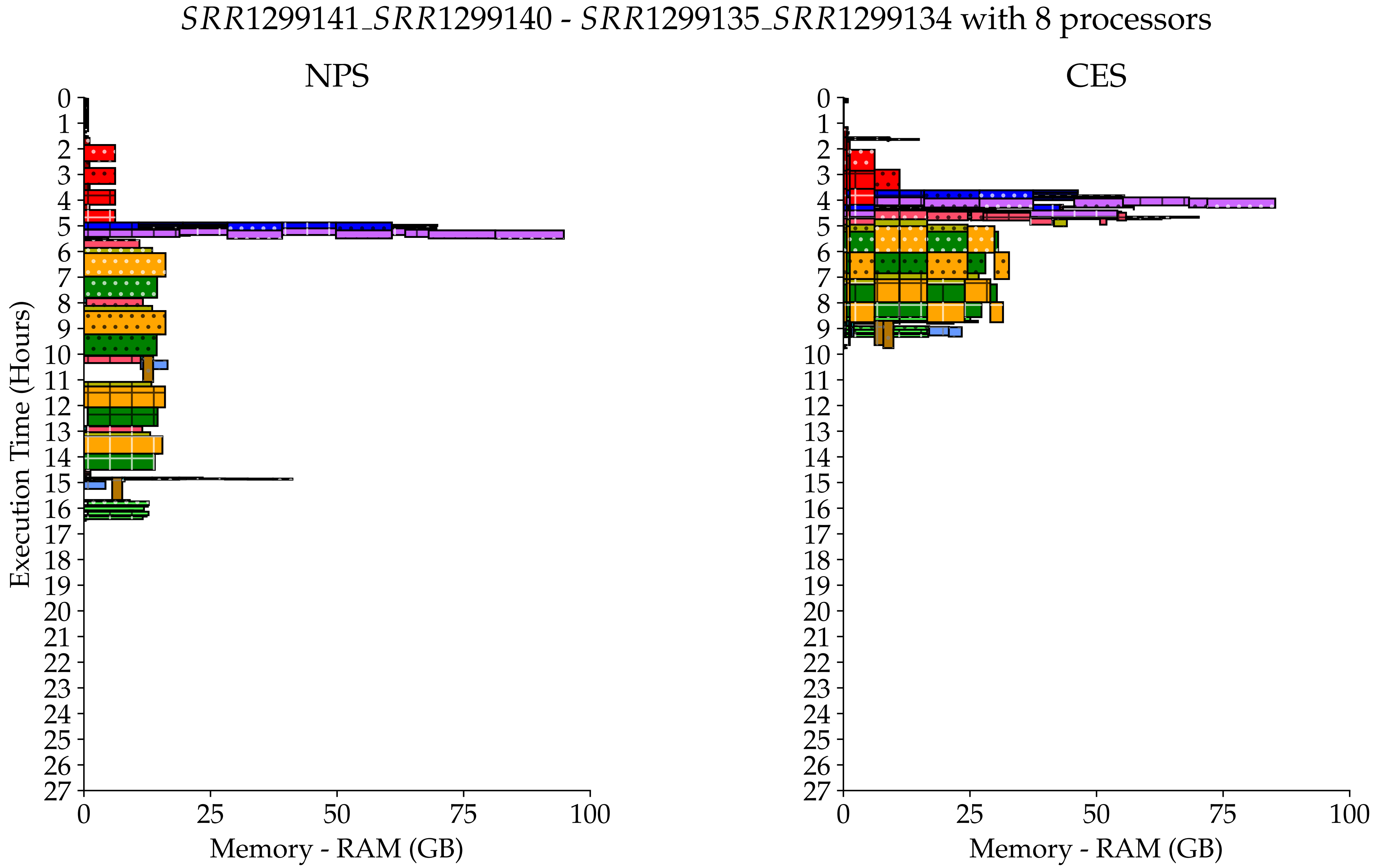
$SRR1299141\_SRR1299140$ - $SRR1299135\_SRR1299134$ with 8 processors
NPS
CES
Execution Time (Hours)
Memory - RAM (GB)
Memory - RAM (GB)

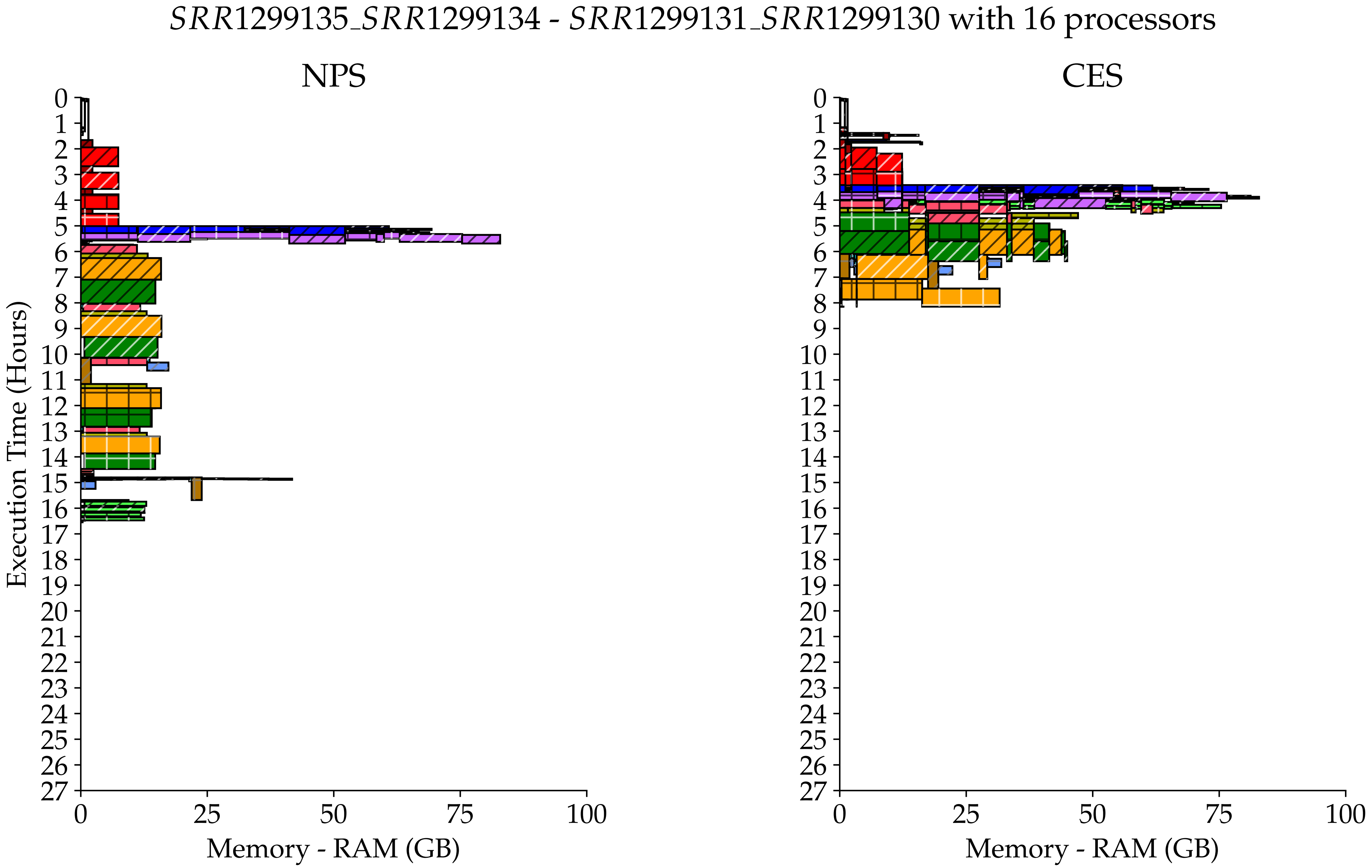

$SRR1299135\_SRR1299134$ - $SRR1299131\_SRR1299130$ with 16 processors
NPS
CES
Execution Time (Hours)
Memory - RAM (GB)
Memory - RAM (GB)
0
25
50
75
100

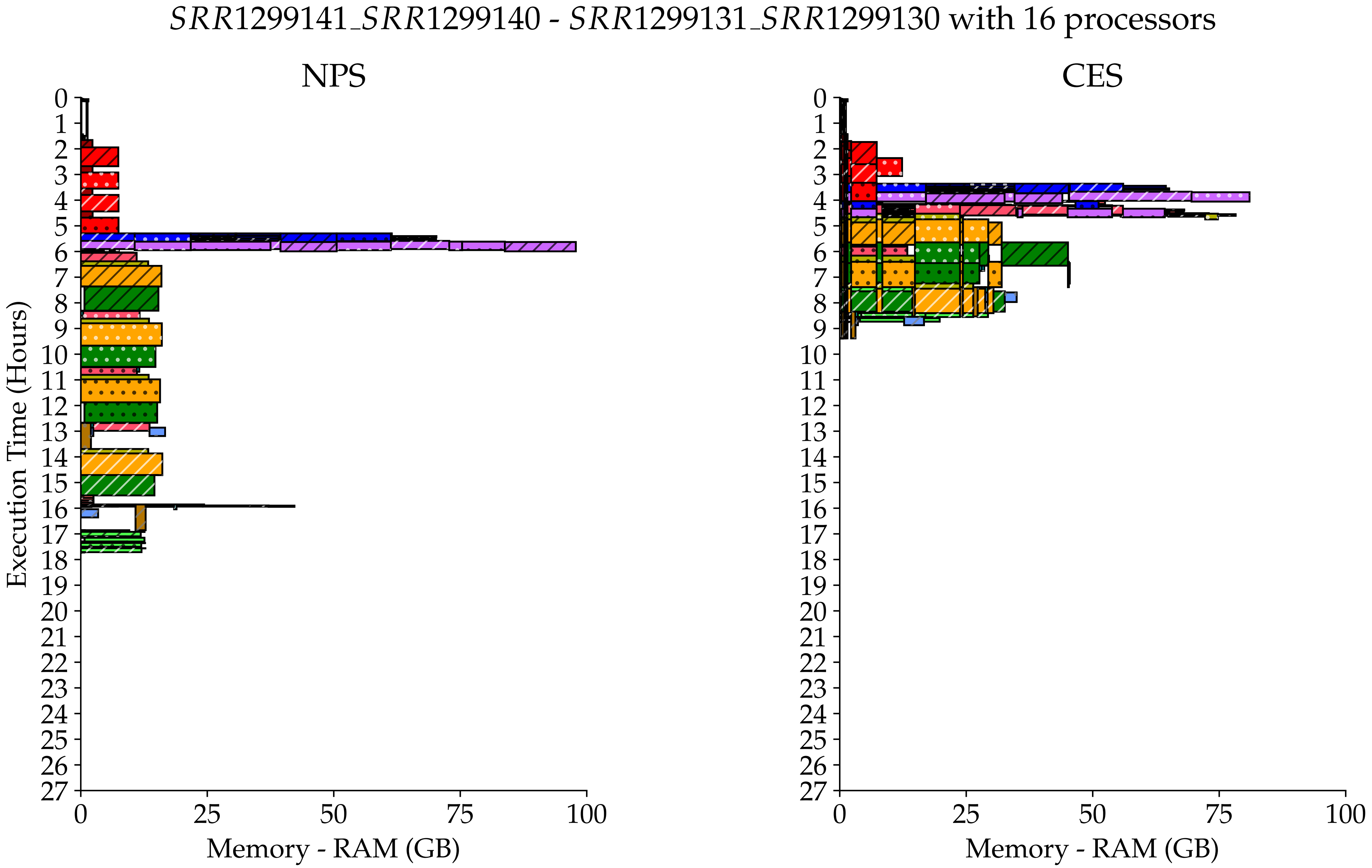

SRR1299141_SRR1299140 - SRR1299131_SRR1299130 with 16 processors
NPS
CES
Execution Time (Hours)
Memory - RAM (GB)
Memory - RAM (GB)

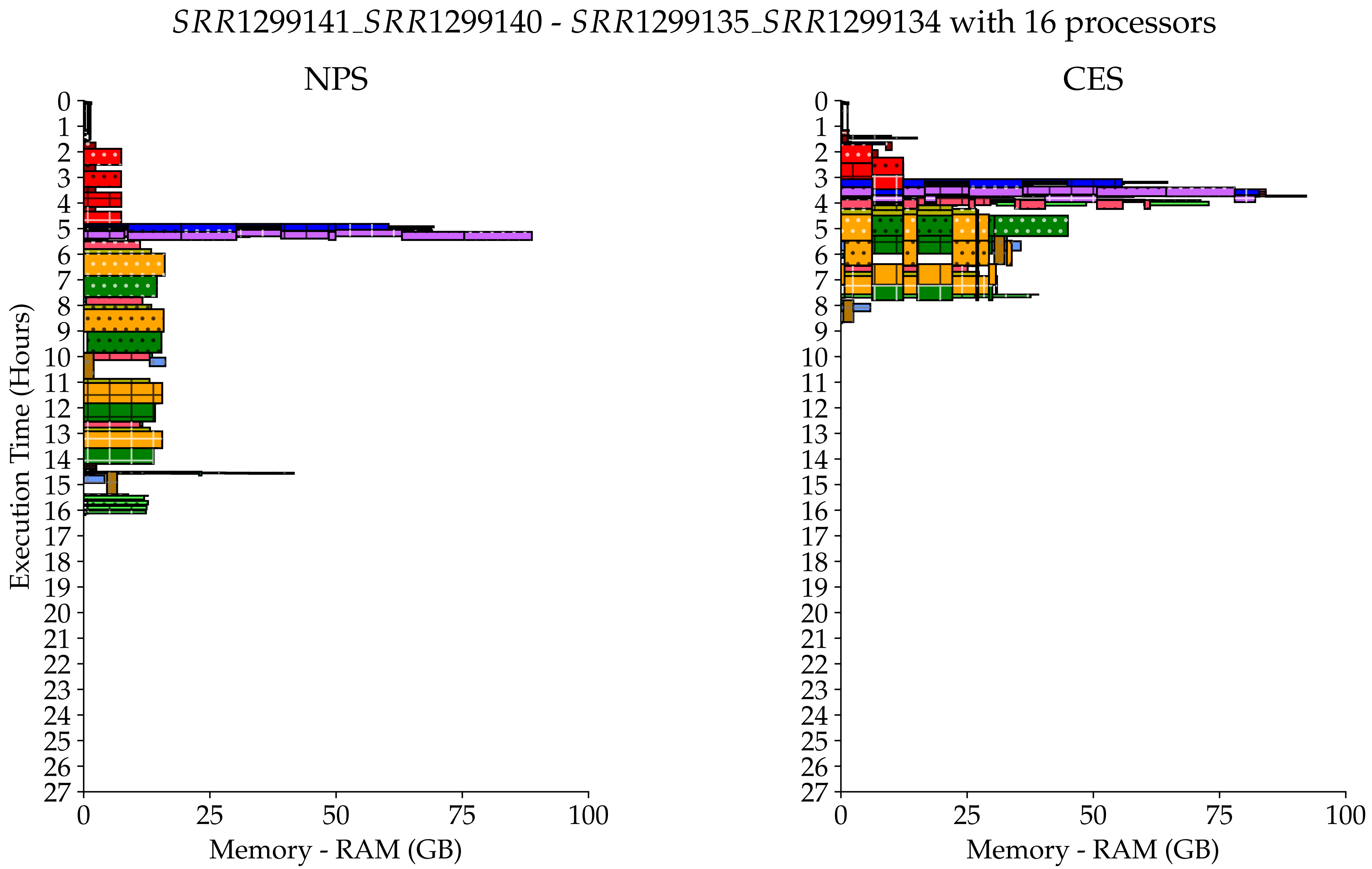
$SRR1299141\_SRR1299140$ - $SRR1299135\_SRR1299134$ with 16 processors
NPS
CES
Execution Time (Hours)
Memory - RAM (GB)
0
25
50
75
100
